# A High-Throughput UHPLC-QqQ-MS Method for Polyphenol Profiling in Rosé Wines

**DOI:** 10.3390/molecules20057890

**Published:** 2015-04-30

**Authors:** Marine Lambert, Emmanuelle Meudec, Arnaud Verbaere, Gérard Mazerolles, Jérémie Wirth, Gilles Masson, Véronique Cheynier, Nicolas Sommerer

**Affiliations:** 1 INRA, UMR 1083 Sciences Pour l’Oenologie, Polyphenol Analysis Facility, 2 place Viala, F-34060 Montpellier, France; E-Mails: eniram.lambert@laposte.net (M.L.); meudec@supagro.inra.fr (E.M.); verbaere@supagro.inra.fr (A.V.); pfp@supagro.inra.fr (G.M.); wirth.clap@wanadoo.fr (J.W.); cheynier@supagro.inra.fr (V.C.); 2 Centre de Recherche et d’Expérimentation sur le Vin Rosé, 70 avenue Wilson, F-83550 Vidauban, France; E-Mail: gilles.masson@vignevin.com

**Keywords:** rosé wine, polyphenols, UHPLC, Multiple Reaction Monitoring—MRM

## Abstract

A rapid, sensitive and selective analysis method using Ultra High Performance Liquid Chromatography coupled to triple-quadrupole Mass Spectrometry (UHPLC-QqQ-MS) has been developed for the quantification of polyphenols in rosé wines. The compound detection being based on specific MS transitions in Multiple Reaction Monitoring (MRM) mode, the present method allows the selective quantification of up to 152 phenolic and two additional non-phenolic wine compounds in 30 min without sample purification or pre-concentration, even at low concentration levels. This method was repeatably applied to a set of 12 rosé wines and thus proved to be suitable for high-throughput and large-scale metabolomics studies.

## 1. Introduction

Rosé is a highly diversified type of wine widely produced and consumed worldwide. Polyphenols are key components of wines, as they are responsible for colour, taste and ultimately the quality of the wines. They constitute a widespread class of plant metabolites which can be found in grapes and can be divided into several families, for instance benzoic acids, hydroxycinnamic acids, stilbenes, flavonols, flavan-3-ols, dihydroflavonols and anthocyanins. Their occurrence in wine depends on several factors such as grape variety, wine-making process, geographic origin and age of the wine. Contrary to red wine, rosé wine polyphenolic composition has not been widely investigated and only a few studies have been published so far [1,2,3]. (Ultra) High Performance Liquid Chromatography ((U)HPLC) coupled to Diode Array Detection (DAD) and/or Mass Spectrometry (MS) detection has been widely used for polyphenol determination in all kinds of matrices (wine [1,2,3,4,5,6,7,8,9,10,11,12,13,14,15,16,17,18,19,20,21,22,23,24], beer [25], fruits and vegetables [22,26,27], tea [14,21,28,29], cocoa [30,31], olive oil [32], plasma [33], urine [34]). Analytical techniques used for food and/or wine polyphenolic analysis have been reviewed [7,35]. The authors detailed several separation techniques used for the fractionation and identification of each class of food polyphenols such as HPLC with UV-Visible or fluorescence detection, often hyphenated to MS detection, (High-Speed) Counter-Current Chromatography ((HS)CCC), Nuclear Magnetic Resonance (NMR) and capillary electrophoresis, sometimes coupled to MS detectors. In both reviews, the authors concluded that LC-MS methods are best suited to identification and routine quantification analyses, tandem MS being highly relevant for structural elucidation and quantification of low-level constituents.

The first polyphenol composition studies resorted to HPLC-DAD methods, for which compound quantification was based on peak areas at selected wavelengths. For instance, Dugo *et al.* [8] reported the quantification of 13 phenolic compounds by two-dimensional HPLC-DAD in 60 min. Wirth *et al.* [3] used a 55-min gradient elution program for the quantitative analysis of 28 polyphenols in rosé wine. Up to 48 phenolic compounds were quantified in red wine by Gomez-Alonso *et al.* [13], however they had to resort to both UV and fluorescence detections and to a ternary solvent chromatographic gradient of 86 min.

Analysis times could be shortened, at the expense of the diversity of the quantified polyphenols. Puértolas *et al.* [2] focused on the quantitative analysis of 14 anthocyanins in rosé wines by HPLC-DAD in a 100-min run time. *trans*-Resveratrol concentrations were determined by Bravo *et al.* [4] by HPLC-DAD (also coupled to fluorescence and electrochemical detections) in a 30-min chromatographic separation following prior sample extraction and concentration.

The use of mass spectrometry coupled to (U)HPLC-DAD techniques has brought significant improvements in polyphenol quantification, mainly by reducing analysis times and increasing sensitivity. However, most methods applied to polyphenol sub-classes analysed separately. The use of triple quadrupole-mass spectrometry (QqQ-MS) detection was relevantly described for the rapid analysis of procyanidins and anthocyanins [22,33]. Ortega *et al.* [30] showed that the use of UHPLC rather than HPLC allowed them to reduce the analysis time from 50 min to 12.5 min for cocoa samples, a less complex matrix mainly composed of procyanidins and alkaloids. Stilbene quantification has been improved regarding the analysis time and the number of quantified analytes [5,25]. Hydroxycinnamic acids were also quantified in red wines by Buiarelli *et al.* [6] by HPLC-QqQ-MS with still a rather long chromatographic separation of 70 min. Gruz *et al.* [14] reported the analysis of 17 phenolic acids by UHPLC-QqQ-MS in 11 min in beverages *i.e.*, white wines, grapefruit juices and green tea infusions. No sample pre-concentration was required for this method, but different purification steps were necessary. A comparative study performed by Spáčil *et al.* [21] on the quantitative analysis in grape, wines and tea of 34 phenolic substances divided into four categories highlights the efficiency of the use of QqQ-MS and UHPLC. Nevertheless, these four classes of compounds were analysed separately. HPLC-DAD coupled to an Ion Trap-Mass Spectrometer (IT-MS) was also used [36] for flavonoid identification, but analysis time remained long considering the number of compounds analysed (11 flavonoid aglycones detected in 70 min).

Still, some more extensive and useful polyphenolic analysis studies have been carried out: Jaitz *et al.* [16] quantified 11 polyphenolic compounds from red wines in less than 13 min by HPLC-QqQ-MS. Twenty eight polyphenols (four procyanidins, 10 phenolic acids and 14 flavones) were quantified in cocoa extracts by HPLC-QqQ-MS in 20 min (Ortega *et al.* [31]). Vaclavik *et al.* [23] resorted to untargeted UHPLC-Q-TOF-MS for establishing a classification of red wines. Although 25 markers were identified in a 24-min gradient time run twice (in positive and negative modes), these compounds were not specifically detected nor quantified. The Mattivi group developed a method focusing on 92 anthocyanins and pigments in red wine after two preliminary semi-preparative chromatography steps (Arapitsas *et al.* [37]) that was recently applied to grape (C. Ehrhardt *et al*. [38]). This group also developped an untargeted method that allowed the detection of 57 polyphenols (among 132 searched polyphenols) in filtered red wine with a 15 min gradient UHPLC-QqQ-MS analysis (U. Vrhovsek *et al.* [39]).

Aware of all these advances in polyphenol composition analyses, a review about analytical techniques applied to wine analysis [7] points out the wine polyphenols analysis requires several specific methods due to the complexity of wine phenolics. The present work challenges this conclusion by describing a high-throughput UHPLC-QqQ-MS quantification method for rosé wine polyphenol analysis. This method was based on Multiple Reaction Monitoring (MRM) detection mode in order to increase both selectivity and sensitivity while reducing analysis times. The suitability of this method for large-scale wine analyses and metabolomics studies was demonstrated on a set of 12 rosé wines.

## 2. Results and Discussion

### 2.1. Choice of the MRM Transitions

Twelve rosé wines were selected according to their geographic origin, their colour and their grape varieties so as to obtain a set as diverse as possible. These wines were first analysed by untargeted UHPLC-Ion Trap (IT)-MS for compound identification.

Low-concentrated compounds which were not detected by UHPLC-IT-MS were specifically targeted by UHPLC-QqQ-MRM based on transitions reported in literature. This was the case for anthocyanin ethyl-bridged proanthocyanidins [27], flavanol-anthocyanin adducts [3,20], caftaric-anthocyanins adducts [3] and anthocyanin derived pigments *i.e.*, pyranoanthocyanins [3,9,10,15,40,41,42] and their flavanol adducts [43]. Once the compounds identified, MRM transitions parameters for each compound were optimised on the UHPLC-QqQ-MS system and compared to data reported in the literature [5,6,14,16,17,22,25,26,28,29,30,31,32,33,34,35,44,45,46].

The MRM transitions were chosen either for a neutral fragment loss or an ion fragment generic to a class of polyphenols, the compound specificity being given by the parent ion mass. Each compound was detected with one quantification transition (quantifier ion) and, when available, one or two confirmation transitions (qualifier ions, see Table 1) to assess detection and quantification specificities. Moreover, the quantification transition has to produce an intense quantifier ion, so as to obtain high signal-to-noise ratios and thus reach low LOQ. When reference standards were available, the MRM transition parameters were optimised by using the Intellistart tool of the MassLynx software which consisted in automatically detecting the major fragments and optimizing cone voltages and collision energies. For other compounds, these parameters were optimised directly on wine, grape juice and Oligomeric ProCyanidins (OPC) powder samples or on enriched extracts (for anthocyanin 3-O-caffeoyl-glycosides). The optimised MRM transition parameters (ion mode, precursor and product ions, retention times) are summarized in Table 1.

Acids were characterized by the loss of the carboxylic acid group (-COO; −44 Th). For acids with a methoxy moiety, such as syringic acid and ferulic acid, the COO loss was also accompanied (or replaced) by the loss of the methyl group (-CH_3_; −15 Th; -COO and -CH_3_; −59 Th). Vanillic acid displayed a −76 Th loss corresponding to the carboxylic acid group and the -OCH_3_ moiety. The loss of the carboxylic acid group together with a 28 a.m.u. group (probably a –CO or an ethyl moiety formed after a rearrangement) was additionally observed for protocatechuic acid and p-coumaric acid. Gallic acid also lost H_2_O in addition to the loss of the carboxylic acid group and of the 28 a.m.u. moiety. Grape Reaction Product (GRP), caftaric, coutaric and fertaric acids were distinguished by their tartrate fragment (149 Th), the latter three ones being characterized by their fragmentation into caffeic, p-coumaric and ferulic acids respectively (which could further undergo -COO and -CH_3_ losses). Ethylic esters of benzoic and hydroxycinnamic acids displayed a −28 a.m.u. loss corresponding to an ethylene group, and a −73 a.m.u. loss representing the –CO-OCH_2_CH_3_ group.

Cis- and trans-resveratrol were characterized by the loss of a phenol group (−94 a.m.u.), and by the formation of a hydroxytropylium ion (*m*/*z* 107 Th), and so was viniferin. *Cis*- and *trans*-piceid lost their glucose entity to form *cis*- and *trans*-resveratrol, which underwent further fragmentation as described.

Non-aglycone flavonols detection was based on the loss of their glucoside (−162 Th) or glucuronide (−176 Th) group. The main fragment (*m*/*z* 153 Th) of quercetin aglycone was the result of a Retro-Diels-Alder (RDA) fragmentation, while the second fragment corresponded to the [M+H-H_2_O-2CO]^+^ ion.

Flavan-3-ols were mainly characterized by fragments resulting from RDA fragmentation (−152 Th), oligomeric and ethyl-bridged proanthocyanidins being also characterized by the loss of monomeric entities.

Taxifolin was qualified by its RDA fragment at *m*/*z* 153 Th, and astilbin by the loss of its rhamnose entity (−146 Th). Anthocyanin detection was mostly based on the loss of the (acetyl-/coumaroyl-/caffeoyl-)glucose entity (acetyl-glucose: −204 Th loss, coumaroyl-glucose: −308 Th loss, caffeoyl-glucose: −324 Th loss, glucose: −162 Th loss), anthocyanins 3,5-diglucosides undergoing the loss of two glucose entities. (epi)catechin-(ethyl-)anthocyanins adducts were also characterized by the additional loss of the (epi)catechin entity.

**Table 1:** List of quantified compounds, MRM parameters (ion mode, precursor and product ions *m*/*z*, retention times), and calibration ranges.

		Ion Mode	*m*/*z* Precursor Ion ^a^ (Th)	*m*/*z* Quantifier ^a^ (Th)	*m*/*z* Qualifiers ^a^ (Th)	Retention Time (min)	Calibration Range (mg·L^−1^)
Benzoic acids and derivatives	*Gallic acid*	-	168.9	125.0	79.0	1.58	0.01–90
	*Protocatechuic acid*	-	152.9	109.0	80.9	3.08	0.01–20
	*Syringic acid*	+	199.0	140.1	155.1	9.98	0.01–20
	*Vanillic acid*	+	169.0	125.0	93.0	7.29	0.01–20
	*Ethyl gallate*	-	197.0	124.0	169.0	10.65	0.01–20
	*Ethyl protocatechuate*	-	181.0	108.0	153.0	13.43	0.01–50
Hydroxycinnamic acids and derivatives	*Caffeic acid*	-	179.0	135.0	79.0	7.69	0.01–50
	*p-coumaric acid*	-	163.0	119.0	91.0	10.71	0.01–20
	*Ferulic acid*	-	193.0	134.0	178.0; 149.0	12.29	0.01–20
	*Caftaric acid (cis- and trans- isomers)*	-	311.0	149.0	179.0; 135.0	4.30; 4.94	0.01–100
	*Coutaric acid (cis- and trans- isomers)*	-	295.1	163.1	149.1; 119.1	6.48; 6.81	as equivalents of *trans-*caftaric acid
	*Fertaric acid*	-	325.1	193.0	134.0; 149.2	9.25	as equivalents of *trans-*caftaric acid
	*GRP (cis- and trans- isomers)*	-	616.0	149.1	167.0	6.13; 7.52	as equivalents of *trans-*caftaric acid
	*Ethyl caffeate*	-	207.1	135.0	179.0; 133.0	18.97	0.01–30
	*Ethyl coumarate*	-	191.0	117.1	163.0	22.35	as equivalents of Ethyl caffeate
Stilbenes	*trans-resveratrol*	+	229.0	135.0	107.1	15.43	0.01–20
	*cis-resveratrol*	+	229.0	135.0 + 107.1 ^b^	135.0; 107.1	18.22	as equivalents of *trans-*resveratrol
	*viniferin (2 isomers)*	+	455.1	361.2	107.1	13.92; 15.33	as equivalents of *trans-*resveratrol
	*trans-piceid*	+	391.1	229.1	135.0	12.87	0.01–20
	*cis-piceid*	+	391.1	229.1 + 135.0 ^b^	229.1; 135.0	15.57	as equivalents of *trans-*piceid
Flavonols	*Quercetin Glc* ^c^	+	465.2	303.0	84.9	15.54	0.01–30
	*Myricetin Glc*	+	481.2	319.1	123.1	13.52	as equivalents of Quercetin Glc
	*Myricetin glucuronide*	+	495.2	319.1	84.9	13.34	as equivalents of Quercetin Glc
	*Quercetin glucuronide*	+	479.2	303.1	85.1; 153.1	15.21	as equivalents of Quercetin Glc
	*Quercetin*	+	303.0	153.1	229.1	21.51	0.01–10
Flavan-3-ols	*Catechin*	+	291.1	139.0	123.1; 165.1	6.95	0.01–50
	*Galloylated dimer*	+	731.2	127.0	139.0; 123.0	11.29	as equivalents of Catechin
	*Trimer-1 (1 isomer)*	+	867.3	127.1	579.2; 289.1	3.47	as equivalents of Catechin
	*Trimers-2 (several isomers)*	+	867.3	127.1	579.2; 289.1	5.5-14.0	as equivalents of Catechin
	*(epi)cat-ethyl-(epi)cat-1 (1 isomer)* ^d^	+	607.1	317.2	165.1	13.91	as equivalents of Catechin
	*(epi)cat-ethyl-(epi)cat-2 (2 co-eluted isomers)*	+	607.1	317.2	165.1	21.37	as equivalents of Catechin
	*Epicatechin*	+	291.1	139.1	123.1; 165.1	10.84	0.01–50
	*Dimer B2*	+	579.2	127.0	139.1; 289.1	9.89	0.01–15
	*Dimer B1*	+	579.2	127.0	139.1; 289.1	6.74	as equivalents of Dimer B2
	*Dimer B3*	+	579.3	127.1	139.1; 289.2	6.21	as equivalents of Dimer B3
	*Dimer B4*	+	579.2	127.0	139.1; 289.1	8.20	as equivalents of Dimer B2
Dihydroflavonols	*Astilbin*	+	451.1	305.1	85.0; 147.1	14.18	as equivalents of Quercetin Glc
	*Taxifolin*	+	305.0	259.1	153.0	12.28	0.01–20
Anthocyanins	*Malvidin 3,5-diGlc*	+	655.2	331.2	493.2	11.87	0.01–30
	*Delphinidin 3,5-diGlc*	+	627.2	303.2	465.2	9.74	as equivalents of Malvidin 3,5-diGlc
	*Cyanidin 3,5-diGlc*	+	611.2	287.2	449.2	10.71	as equivalents of Malvidin 3,5-diGlc
	*Petunidin 3,5-diGlc*	+	641.2	317.2	479.2	11.13	as equivalents of Malvidin 3,5-diGlc
	*Peonidin 3,5-diGlc*	+	625.2	301.2	463.2	11.70	as equivalents of Malvidin 3,5-diGlc
	*Malvidin 3-O-Glc*	+	493.2	331.2	315.0	13.80	0.01–50
	*Delphinidin 3-O-Glc*	+	465.2	303.1	229.0	11.78	as equivalents of Malvidin 3-*O*-Glc
	*Cyanidin 3-O-Glc*	+	449.2	287.1	137.2	12.37	as equivalents of Malvidin 3-*O*-Glc
	*Petunidin 3-O-Glc*	+	479.2	317.2	302.3	12.87	as equivalents of Malvidin 3-*O*-Glc
	*Peonidin 3-O-Glc*	+	463.2	301.2	286.1	13.40	as equivalents of Malvidin 3-*O*-Glc
	*Delphinidin 3-O-acetyl-Glc*	+	507.2	303.0	229.0	16.04	as equivalents of Malvidin 3-*O*-Glc
	*Cyanidin 3-O-acetyl-Glc*	+	491.2	287.1	137.0	18.33	as equivalents of Malvidin 3-*O*-Glc
	*Petunidin 3-O-acetyl-Glc*	+	521.2	317.2	302.3	19.76	as equivalents of Malvidin 3-*O*-Glc
	*Peonidin 3-O-acetyl-Glc*	+	505.2	301.1	286.2	20.97	as equivalents of Malvidin 3-*O*-Glc
	*Malvidin 3-O-acetyl-Glc*	+	535.2	331.2	315.	21.18	as equivalents of Malvidin 3-*O*-Glc
	*Delphinidin 3-O-coumaroyl-Glc (cis- and trans- isomers)*	+	611.2	303.1	229.1	20.71; 22.05	as equivalents of Malvidin 3-*O*-Glc
	*Cyanidin 3-O-coumaroyl-Glc (cis- and trans- isomers)*	+	595.2	287.1	157.1	21.60; 22.67	as equivalents of Malvidin 3-*O*-Glc
	*Petunidin 3-O-coumaroyl-Glc (cis- and trans- isomers)*	+	625.2	317.2	302.3	21.97; 22.83	as equivalents of Malvidin 3-*O*-Glc
	*Peonidin 3-O-coumaroyl-Glc (cis- and trans- isomers)*	+	609.2	301.2	286.2	22.53; 23.17	as equivalents of Malvidin 3-*O*-Glc
	*Malvidin 3-O-coumaroyl-Glc (cis- and trans- isomers)*	+	639.2	331.2	315.2	22.60; 23.18	as equivalents of Malvidin 3-*O*-Glc
	*Delphinidin 3-O-caffeoyl-Glc*	+	627.2	303.1	569.2	20.21	as equivalents of Malvidin 3-*O*-Glc
	*Cyanidin 3-O-caffeoyl-Glc*	+	611.2	287.0	-	21.27	as equivalents of Malvidin 3-*O*-Glc
	*Petunidin 3-O-caffeoyl-Glc*	+	641.2	317.2	-	21.69	as equivalents of Malvidin 3-*O*-Glc
	*Peonidin 3-O-caffeoyl-Glc*	+	625.2	301.2	286.2	22.35	as equivalents of Malvidin 3-*O*-Glc
	*Malvidin 3-O-caffeoyl-Glc*	+	655.2	331.2	315.1	22.43	as equivalents of Malvidin 3-*O*-Glc
	*(epi)cat-ethyl-peonidin 3-O-Glc (4 isomers)*	+	779.2	327.1	489.1	16.98; 18.38; 20.26; 20.90	as equivalents of Malvidin 3-*O*-Glc
	*(epi)cat-ethyl-malvidin 3-O-Glc (4 isomers)*	+	809.2	357.3	519.1	17.93; 19.46; 20.46 (2 co-eluted isomers)	as equivalents of Malvidin 3-*O*-Glc
	*(epi)cat-ethyl-malvidin 3-O-coumaroyl-Glc (2 co-eluted isomers)*	+	955.2	647.2	357.1	23.31	as equivalents of Malvidin 3-*O*-Glc
	*Delphinidin 3-O-Glc-(epi)cat*	+	753.2	591.2	303.1	8.32	as equivalents of Malvidin 3-*O*-Glc
	*Cyanidin 3-O-Glc-(epi)cat*	+	737.2	575.2	287.1	8.53	as equivalents of Malvidin 3-*O*-Glc
	*Petunidin 3-O-Glc-(epi)cat*	+	767.2	605.2	317.1	10.20	as equivalents of Malvidin 3-*O*-Glc
	*Peonidin 3-O-Glc-(epi)cat*	+	751.2	589.2	301.1	10.98	as equivalents of Malvidin 3-*O*-Glc
	*Malvidin 3-O-Glc-(epi)cat (2 isomers)*	+	781.2	619.2	257.1	11.05; 12.09	as equivalents of Malvidin 3-*O*-Glc
	*Malvidin 3-O-coumaroyl-Glc-(epi)cat (2 isomers)*	+	927.2	619.2	373.2	15.43; 15.96	as equivalents of Malvidin 3-*O*-Glc
	*(epi)cat-delphinidin 3-O-Glc A-F bicyclic*	+	755.2	315.2	593.2	11.13	as equivalents of Malvidin 3-*O*-Glc
	*(epi)cat-cyanidin 3-O-Glc A-F bicyclic*	+	739.2	299.1	587.2	11.78	as equivalents of Malvidin 3-*O*-Glc
	*(epi)cat-petunidin 3-O-Glc A-F bicyclic*	+	769.2	329.1	617.2	12.20	as equivalents of Malvidin 3-*O*-Glc
	*(epi)cat-peonidin 3-O-Glc A-F bicyclic*	+	753.2	313.1	601.2	12.87	as equivalents of Malvidin 3-*O*-Glc
	*(epi)cat-malvidin 3-O-Glc A-F bicyclic*	+	783.2	343.1	469.2	13.14	as equivalents of Malvidin 3-*O*-Glc
	*Caftaric-peonidin 3-O-Glc (2 co-eluted isomers)*	+	773.2	611.2	151.0	11.07	as equivalents of Malvidin 3-*O*-Glc
	*Caftaric-malvidin 3-O-Glc (2 co-eluted isomers)*	+	803.2	641.2	181.1	11.62	as equivalents of Malvidin 3-*O*-Glc
	*Coutaric-malvidin 3-O-Glc (2 co-eluted isomers)*	+	787.2	625.2	-	12.44	as equivalents of Malvidin 3-*O*-Glc
	*Pyranodelphinidin 3-O-Glc*	+	489.2	327.2	-	12.19	as equivalents of Malvidin 3-*O*-Glc
	*Pyranocyanidin 3-O-Glc*	+	473.2	311.2	-	13.04	as equivalents of Malvidin 3-*O*-Glc
	*Pyranopetunidin 3-O-Glc*	+	503.2	341.1	-	13.69	as equivalents of Malvidin 3-*O*-Glc
	*Pyranopeonidin 3-O-Glc*	+	487.2	325.1	310.2	14.67	as equivalents of Malvidin 3-*O*-Glc
	*Pyranomalvidin 3-O-Glc (vitisin B)*	+	517.2	355.1	339.2	15.55	as equivalents of Malvidin 3-*O*-Glc
	*Carboxypyranodelphinidin 3-O-Glc*	+	533.2	371.2	-	12.36	as equivalents of Malvidin 3-*O*-Glc
	*Carboxypyranocyanidin 3-O-Glc*	+	517.2	355.2	-	13.26	as equivalents of Malvidin 3-*O*-Glc
	*Carboxypyranopetunidin 3-O-Glc*	+	547.2	385.2	271.2	13.80	as equivalents of Malvidin 3-*O*-Glc
	*Carboxypyranopeonidin 3-O-Glc*	+	531.2	369.2	297.2	14.97	as equivalents of Malvidin 3-*O*-Glc
	*Carboxypyranomalvidin 3-O-Glc (vitisin A)*	+	561.2	399.1	383.1	15.87	as equivalents of Malvidin 3-*O*-Glc
	*Pyranodelphinidin 3-O-acetyl-Glc*	+	531.2	327.2	-	12.79	as equivalents of Malvidin 3-*O*-Glc
	*Pyranocyanidin 3-O-acetyl-Glc*	+	515.2	311.2	-	13.91	as equivalents of Malvidin 3-*O*-Glc
	*Pyranopetunidin 3-O-acetyl-Glc*	+	545.2	341.2	-	14.50	as equivalents of Malvidin 3-*O*-Glc
	*Pyranopeonidin 3-O-acetyl-Glc*	+	529.2	325.2	309.2	15.97	as equivalents of Malvidin 3-*O*-Glc
	*Pyranomalvidin 3-O-acetyl-Glc*	+	559.2	355.2	339.2	16.67	as equivalents of Malvidin 3-*O*-Glc
	*Carboxypyranodelphinidin 3-O-acetyl-Glc*	+	575.2	371.2	283.2	12.62	as equivalents of Malvidin 3-*O*-Glc
	*Carboxypyranocyanidin 3-O-acetyl-Glc*	+	559.2	355.2	527.2	13.91	as equivalents of Malvidin 3-*O*-Glc
	*Carboxypyranopetunidin 3-O-acetyl-Glc*	+	589.2	385.2	-	14.34	as equivalents of Malvidin 3-*O*-Glc
	*Carboxypyranopeonidin 3-O-acetyl-Glc*	+	573.2	369.2	281.2	16.28	as equivalents of Malvidin 3-*O*-Glc
	*Carboxypyranomalvidin 3-O-acetyl-Glc*	+	603.2	399.2	383.2	16.90	as equivalents of Malvidin 3-*O*-Glc
	*Pyranopeonidin 3-O-coumaroyl-Glc*	+	633.2	325.2	310.2	21.69	as equivalents of Malvidin 3-*O*-Glc
	*Pyranomalvidin 3-O-coumaroyl-Glc*	+	663.2	355.2	-	21.78	as equivalents of Malvidin 3-*O*-Glc
	*Carboxypyranopetunidin 3-O-coumaroyl-Glc*	+	693.2	385.2	370.2	19.13	as equivalents of Malvidin 3-*O*-Glc
	*Carboxypyranopeonidin 3-O-coumaroyl-Glc*	+	677.2	369.2	354.1	21.37	as equivalents of Malvidin 3-*O*-Glc
	*Carboxypyranomalvidin 3-O-coumaroyl-Glc*	+	707.2	399.0	383.0	21.44	as equivalents of Malvidin 3-*O*-Glc
	*p-hydroxyphenylpyranopeonidin 3-O-Glc*	+	579.2	417.2	402.2	23.52	as equivalents of Malvidin 3-*O*-Glc
	*p-hydroxyphenylpyranomalvidin 3-O-Glc*	+	609.2	447.2	431.1	23.56	as equivalents of Malvidin 3-*O*-Glc
	*p-hydroxyphenylpyranopeonidin 3-O-acetyl-Glc*	+	621.2	417.2	-	23.59	as equivalents of Malvidin 3-*O*-Glc
	*p-hydroxyphenylpyranomalvidin 3-O-acetyl-Glc*	+	651.2	447.2	431.2	23.63	as equivalents of Malvidin 3-*O*-Glc
	*p-hydroxyphenylpyranopeonidin 3-O-coumaroyl-Glc*	+	725.2	417.2	-	23.67	as equivalents of Malvidin 3-*O*-Glc
	*p-hydroxyphenylpyranomalvidin 3-O-coumaroyl-Glc*	+	755.2	447.1	431.1	23.68	as equivalents of Malvidin 3-*O*-Glc
	*Catechylpyranopeonidin 3-O-Glc*	+	595.2	433.2	418.1	23.39	as equivalents of Malvidin 3-*O*-Glc
	*Catechylpyranomalvidin 3-O-Glc (pinotin A)*	+	625.2	463.2	447.2	23.44	as equivalents of Malvidin 3-*O*-Glc
	*Catechylpyranopetunidin 3-O-acetyl-Glc*	+	653.2	449.2	-	23.59	as equivalents of Malvidin 3-*O*-Glc
	*Catechylpyranopeonidin 3-O-acetyl-Glc*	+	637.2	433.2	-	23.47	as equivalents of Malvidin 3-*O*-Glc
	*Catechylpyranomalvidin 3-O-acetyl-Glc*	+	667.2	463.2	-	23.51	as equivalents of Malvidin 3-*O*-Glc
	*Catechylpyranopetunidin 3-O-coumaroyl-Glc*	+	757.2	449.2	-	23.67	as equivalents of Malvidin 3-*O*-Glc
	*Catechylpyranopeonidin 3-O-coumaroyl-Glc*	+	741.2	433.2	-	23.55	as equivalents of Malvidin 3-*O*-Glc
	*Catechylpyranomalvidin 3-O-coumaroyl-Glc*	+	771.2	463.2	-	23.59	as equivalents of Malvidin 3-*O*-Glc
	*Guaiacylpyranomalvidin 3-O-Glc*	+	639.2	477.2	462.2	23.60	as equivalents of Malvidin 3-*O*-Glc
	*Guaiacylpyranomalvidin 3-O-acetyl-Glc*	+	681.2	477.2	-	23.63	as equivalents of Malvidin 3-*O*-Glc
	*Guaiacylpyranomalvidin 3-O-coumaroyl-Glc*	+	785.2	477.1	461.3	23.71	as equivalents of Malvidin 3-*O*-Glc
	*Syringylpyranomalvidin 3-O-Glc*	+	669.2	507.2	-	23.59	as equivalents of Malvidin 3-*O*-Glc
	*Petunidin 3-O-acetyl-Glc-(epi)cat*	+	809.2	359.1	605.2; 317.1	13.05	as equivalents of Malvidin 3-*O*-Glc
	*Peonidin 3-O-acetyl-Glc-(epi)cat*	+	793.2	343.1	589.2; 301.1	13.22	as equivalents of Malvidin 3-*O*-Glc
	*Malvidin 3-O-acetyl-Glc-(epi)cat*	+	823.2	373.2	331.1; 619.2	13.49	as equivalents of Malvidin 3-*O*-Glc
	*Pyranopeonidin 3-O-Glc-(epi)cat*	+	775.2	613.2	461.2	23.19	as equivalents of Malvidin 3-*O*-Glc
	*Pyranomalvidin 3-O-Glc-(epi)cat*	+	805.2	491.0	643.2	23.43	as equivalents of Malvidin 3-*O*-Glc
	*Pyranomalvidin 3-O-coumaroyl-Glc-(epi)cat*	+	951.2	643.2	-	23.43	as equivalents of Malvidin 3-*O*-Glc
	*Unknown "551" ^e^*	+	551.2	221.2	151.1	13.8	as equivalents of Malvidin 3-*O*-Glc
	*Unknown "581"* ^e^	+	581.2	251.2	165.1	14.43	as equivalents of Malvidin 3-*O*-Glc
	*Unknown "1095"* ^e^	+	1095.2	771.2	302.1	22.35	as equivalents of Malvidin 3-*O*-Glc
	*Unknown "1099"* ^e^	+	1099.2	937.2	-	21.18	as equivalents of Malvidin 3-*O*-Glc
Others (aminoacids and alcohols)	*Tyrosine*	+	182.0	136.1	165.1	1.61	0.01–50
	*Tyrosol* ^f^	+	121.0	77.0	93.1	5.3	0.01–50
	*Hydroxytyrosol*	+	155.0	91.0	81.0	3.61	0.01–20
	*Tryptophol*	+	162.0	127.2	117.0	12.36	0.01–20
	*Tryptophan*	-	203.0	116.0	74.0	5.98	0.01–50

^a^ span: 0.2; ^b^ The *cis*-isomers of resveratrol and piceid were quantified by integrating a sum of two transition signals, as explained in part 2.1; ^c^ Glc: glucoside; ^d^ epi(cat): catechin or epicatechin; ^e^ The reasons for the quantification of the unknown compounds as equivalents of malvidin 3-*O*-glucoside are presented in part 2.2; ^f^ The precursor ion *m*/*z* for tyrosol does not refer to the [M+H]^+^ ion, but to the [M-H_2_O+H]^+^ ion due to in-source fragmentation, even under mild ionization conditions [45].

Tyrosol was characterized by two fragments: *m*/*z* 77 Th and *m*/*z* 93 Th corresponding to the aromatic ring and the phenol group, respectively. Tryptophan’s detection was based on the fragment at *m*/*z* 116 Th representing the indole ion of the molecule obtained by the loss of the side-chain, and the fragment at *m*/*z* 74 Th representing the [CHNH_2_-COO]^−^ ion. Tyrosine was characterized by the formation of an immonium ion and by the loss of a -NH_3_ group.

Polyphenols are a wide family of natural compounds, therefore the major part of them are not commercially available, and their deuterated forms even less so. For that reason, an absolute quantification was not possible, and these compounds were quantified as equivalents of reference standards from the same chemical class, as specified in Table 1. Such a relative quantification method is a suitable approach since the current UHPLC-QqQ-MS method is intended to large-scale comparative studies. Several particular cases were pointed out. The *cis*-resveratrol fragmentation pathway is quantitatively different from the one of the *trans*-isomer. Quantifying *cis*-resveratrol as equivalents of *trans*-resveratrol, based on the major transition (or even a minor one), was thus not appropriate, by comparison to UV-based quantification [47]. It appeared that quantifying *cis*-resveratrol as equivalents of *trans*-resveratrol by integrating the sum of both selected transition traces instead of only one of them matched to the UV-based quantification. This method was verified on several wine samples spiked with different amounts of resveratrol (data not shown). This statement was also applied by extension to the quantification of *cis*-piceid (the glucoside form of resveratrol) as equivalents of *trans*-piceid. Ethyl coumarate was not commercially available and was quantified as equivalents of ethyl caffeate. However, this quantification was not based on the main transition of ethyl caffeate (Electrospray Ionization (ESI) ESI + 207 > 135 Th) but on a minor one (ESI + 207 > 133 Th; −74 Th loss) so as to match to the main fragmentation of ethyl coumarate (ESI + 191 > 117 Th; −73 Th loss). In the same way, coutaric acid (ESI − 295 > 163 Th) and fertaric acid (ESI − 325 > 193 Th) quantifications were based on a minor, still intense, fragmentation transition of caftaric acid (ESI − 311 > 179 Th) corresponding to the same −132 Th loss. GRP (main fragmentation: ESI − 616 > 149 Th) was quantified by resorting to the major fragmentation of caftaric acid (ESI − 311 > 149 Th) in order to obtain the same fragment. Astilbin, also known as dihydroquercetin 3-*O*-rhamnoside, is not commercially available, and was thus quantified as equivalents of its structurally closest compound, quercetin glucoside, which also loses its sugar moiety.

### 2.2. Unknown Compounds

Four compounds detected by UHPLC-IT-MS were not identified. These compounds were named according to their *m*/*z* ratio in positive mode: “unknown581” and “unknown551”, both consisting in double peaks, “unknown1099” which was detected as doubly charged [550]^2+^, and “unknown1095”. They displayed an absorbance in the anthocyanin wavelength range (around 520 nm) and were characterized by the loss of one glucose entity (−162) for “unknown581” and “unknown551” and two successive glucose entity losses for “unknown1099” and “unknown1095”. The *m*/*z* difference between “unknown 551” and “unknown 581” was also noticeable, since it corresponded to the mass difference between peonidin and malvidin compounds. Although their structure was not elucidated, these unknown compounds were added to the UHPLC-QqQ-MS method because of their potential relevancy for metabolomics’ approaches. They were quantified as equivalents of malvidin 3-*O*-glucoside, as they displayed UV spectra and glucose losses characteristic of anthocyanin compounds. Fragmentation spectra are available as supplementary data.

### 2.3. Optimisation of UHPLC-QqQ-MS Conditions

UHPLC is a technique that allows an enhanced chromatographic resolution compared to classical HPLC, due to an increase in column pressure (up to 1000 bars). The optimal working flow rate is then much higher with UHPLC than with HPLC. This consequence motivated the choice of a 1 mm-diameter column *versus* a 2.1 mm-diameter column for the development of the quantification method described in this paper. The mobile phase flow rate could thus be decreased from 0.5–0.6 mL·min^−1^ to 0.17 mL·min^−1^ in order to suit better to the use of an ESI-MS interface, without degrading the peak resolution, while reducing solvent consumption. The percentage of formic acid is a key parameter for polyphenol analysis, since it has to be high enough for maintaining the anthocyanins in their flavylium cationic form (pH < 2) [48], but not too high so as to avoid signal suppression in the source of the mass spectrometer, especially for acids. Three percentage values (1%, 2% and 5%) were tested for the analysis of several hydroxycinnamic acids. A MS signal intensity loss of 25% and 50% was observed when increasing the percentage of formic acid from 1% to 2% and 5% respectively. The optimal working percentage of formic acid in the mobile phase was thus set at 1%, what corresponded to a pH of 2.1 in solvent A. Methanol as solvent B was preferred to acetonitrile despite its higher viscosity, because an enhancement of the MS signal intensity was observed when using methanol, especially for acids in negative ionization mode [49].

Polyphenolic compounds may be degraded by heat [50], however, increasing the column temperature to 40 °C was necessary to achieve optimal chromatographic resolution by reducing methanol viscosity and thus increasing the mobile phase flow rate.

Once the MRM transitions parameters optimised as described in Table 1, the chromatographic separation was adapted so as to obtain a gradient elution program as short as possible without losing sensitivity. Sensitivity was maintained by adapting dwell times for each compound MS transition so as to obtain between 10 and 15 points per peak. Dwell times ranged from 0.005 s, *i.e.*, the higher scanning speed limitation of the QqQ mass spectrometer used in MRM mode, to 0.705 s, with a mean and median dwell times of 0.077 s and 0.005 s respectively. The higher the number of overlapped MRM functions, the lower the dwell times. Most of the dwell times were very low, which showed the high-throughput character of the method exploiting the full capacities of the mass spectrometer analyser. The wide gap between the mean and the median values illustrated the high heterogeneity of the analysed compounds. This resulted in different elution behaviours, hence a variation in the number of overlapped MRM functions during the chromatographic separation. Some time sequences were characterized by a great number of coeluted compounds, whereas other time sequences were relatively poorer in terms of analysed compounds.

### 2.4. Advantages of the Present Method

One hundred and fifty four (154) compounds were quantified by UHPLC-QqQ-MS within a chromatographic separation of 30 min including column re-equilibration. The wine samples were analysed without prior sample extraction, clean-up or concentration steps.

**Figure 1:**
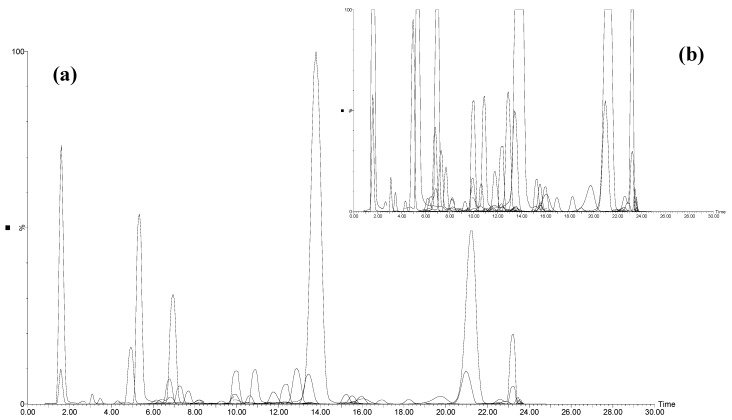
Superimposition of MRM traces from the UHPLC-QqQ-MS analysis of a rosé wine, (**a**) full display; (**b**) six-time zoomed display.

Such a short analysis time could be obtained thanks to the selectivity of the method: the MRM mode focuses on transitions that are specific to the quantified compounds. Compounds which show the same precursor and product ions, *i.e.*, delphinidin 3-*O*-glucoside and quercetin glucoside or anthocyanins 3-*O*-caffeoyl-Glc and anthocyanins 3,5-diGlc, remained separated by their retention times. An example of a chromatogram is shown in Figure 1.

This example highlights the high heterogeneity of the wine sample with some major compounds and a large number of minor compounds. The large dynamic range of the QqQ mass spectrometer allowed the measurement of such concentration differences. The six-time zoomed chromatogram displays a multitude of overlapped MRM peaks, which illustrates the complexity of the sample and the ability of the MRM method to specifically distinguish co-eluted compounds. Improved selectivity is an important advantage compared to HPLC-DAD methods, for which compounds quantification is based on peak areas at selected wavelengths, requiring almost full separation of all compounds, with relatively long gradient elution programs. Nevertheless, the enhancement of selectivity brought by the QqQ-MRM detection did not allow to get rid of all other co-elution problems, in particular matrix effects and ionization competition between co-eluted molecules. For that reason, it was necessary to maintain a minimum chromatographic separation of 30 min while pushing the MRM scan rate of the mass spectrometer to its maximum.

The sensitivity of the present method was largely enhanced by the use of the MRM mode. Its efficiency—sensitivity enhancement and time saving—was increased by the possibility of running both positive and negative ion modes in a single run, contrary to the UHPLC-Q-TOF-MS method applied to red wines by Vaclavik *et al.* [23]. The sensitivity of this method was characterized by very low limits of quantification (LOQ). The LOQ were determined by measuring the noise for each compound, and by converting 10 times these values into concentrations by means of the calibration curves. The concentrations of the different compounds in the 12 analyzed rosé wines with their calculated LOQ are available as supplementary data. The LOQ were different from a wine to another, as the matrix effects were specific to each wine.

Sensitivity combined to selectivity resulted in a major advance in wine polyphenolic composition analysis, since it allowed the quantification of 152 phenolic compounds (and two other non-phenolic wine compounds *i.e.*, tryptophan and tryptophol) *versus* 48 phenolic compounds with the method described by Gomez-Alonso *et al.* [13] and 57 phenolic compounds with the method described by Vrhovsek *et al.* [39]. This is all the more relevant that polyphenolic compounds are present in lesser quantities in rosé wines than in red wines, and thus more difficult to detect and quantify.

Nevertheless, a particular attention should be paid to *trans*-resveratrol, which is partially co-eluted with in-source fragmented *cis-*piceid (a glycosylated form of resveratrol which easily loses its glucose moiety).

### 2.5. Chemometric Analysis—An Illustrative Application of this Method

The present UHPLC-QqQ-MS polyphenols quantification method is particularly suitable for high-throughput wine analyses, especially when it is coupled with chemometrics. Approached in this way, large-scale food metabolomics studies can be performed in order to discriminate between wines samples or connecting their polyphenolic composition to other characteristics.

**Figure 2:**
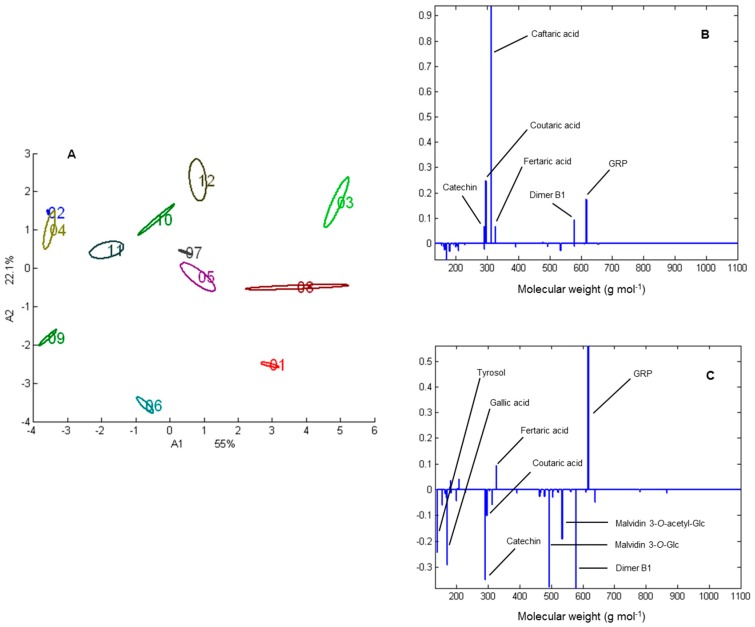
(**A**) PCA scores plot of the wines 1 to 12; (**B**) Loadings associated to the first common component A1; (**C**) Loadings associated to the second common component A2.

To illustrate this potential, we present in Figure 2 the Principal Component Analysis (PCA) of the data table originating from the analysis in triplicate of the 12 roses wines previously selected. Mass data were imported and treated in the Matlab environment (The Mathworks, Inc., Natick, MA, USA). PCA was performed on the variance-covariance matrix, without previous column standardization of the data table.

The plot of the PCA wines scores (Figure 2A) illustrates the variability between the polyphenolic composition of the analysed samples. In comparison, the repeatability of the method, evaluated by confidence ellipses set at 95% around the mean results is quite satisfactory. Figure 2B,C represent the loadings associated to the first and the second principal component, respectively. On these plots, the abscissa axis represents the molecular weight of each analysed compound while the ordinate axis represents its contribution to the spread out of samples on the corresponding component. Thus, for the first axis, the variability between samples of opposite sign mainly address to caftaric acid (higher concentrations measured for samples 1, 3 and 8), followed by coutaric acid, GRP and dimer B1. The second axis is more complex to interpret and is mainly characterized by the contributions of GRP (higher concentration for samples presenting a positive coordinate on the second component) and by the contribution of tyrosol, gallic acid, catechin, malvidin 3-*O*-glucoside, dimer B1 and malvidin 3-*O*-acetylglucoside (higher concentration for samples presenting a negative coordinate).

## 3. Experimental Section

### 3.1. Chemicals

Formic acid and HPLC grade methanol were purchased from VWR Prolabo (Fontenay-sous-Bois, France). Deionized water was obtained from a Milli-Q purification system (Millipore, Molsheim, France). Standards of gallic acid, vanillic acid, caffeic acid and ferulic acid were purchased from Fluka (Buchs, Switzerland). Standards of protocatechuic acid, syringic acid, p-coumaric acid, ethyl protocatechuate, tyrosol, tryptophol, tryptophan, trans-resveratrol, quercetin, catechin, epicatechin and taxifolin were purchased from Sigma-Aldrich (St. Louis, MO, USA). Standards of ethyl gallate, ethyl caffeate, tyrosine, hydroxytyrosol, malvidin 3-*O*-glucoside chloride and malvidin 3,5-diglucoside chloride were purchased from Extrasynthese (Geney, France). Standards of *trans*-caftaric acid, quercetin glucoside and dimer B2 were purchased from Phytolab (Vestenbergsgreuth, Germany). Standard of *trans*-piceid was purchased from Selleckchem (Houston, TX, USA). Oligomeric ProCyanidins (OPC) powder used for method development was supplied by Berkem (Gardonne, France).

### 3.2. Samples

Twelve rosé wines, respectively numbered from 1 to 12, were selected by the Centre du Rosé (Vidauban, France) for their geographic origin diversity and their wide colour range: Mateus, Portugal (sparkling); Palatinat, Germany; Slovakia; Vaud, Switzerland; Latium, Italy; La Rioja, Spain; Provence, France (two wines); Bergerac, France; Languedoc, France; Loire, France; Champagne, France (sparkling).

### 3.3. Standard and Sample Preparation

Standard solutions were prepared in methanol/H2O 20/80 (*v*/*v*). Their calibration ranges are summarized in Table 1. Rosé wines were filtered through 0.45 µm filters (Hydrophobic Durapore, Millipore) under vacuum in order to remove solid particles. The filtration flasks were then stoppered and the vacuum was maintained for 5 min in order to degas the wines. 1 mL of each wine was filtered through a 0.2 µm regenerated cellulose membrane syringe filter (Phenex, Phenomenex, Le Pecq, France), and the samples were injected in triplicates for UHPLC-QqQ-MS analysis.

### 3.4. UHPLC-DAD-IT-MS^n^ Conditions

Preliminary experiments for compound identification were carried out on an Acquity UHPLC system equipped with a diode array detector (Waters, Saint-Quentin-en-Yvelines, France) and hyphenated to an AmaZon X ion-trap mass spectrometer (Bruker Daltonique, Wissembourg, France) with electrospray ionization (ESI) operating in switching positive and negative modes. UHPLC-DAD-ESI-IT-MSn analyses were performed with a Waters ACQUITY chromatography system coupled to a Bruker Amazon X ion-trap mass spectrometer (Acquity UPLC, Waters—AmazonX IT, Bruker Daltonique). The UHPLC system included a binary pump, a cooled autosampler maintained at 7 °C and equipped with a 5-µL sample loop, a 100-µL syringe, a 30-µL needle and a DAD. The Compass HyStar software suite was used to control the instruments and to acquire and process the data. The column used for chromatographic separation was a reversed-phase Acquity HSS T3 1.8 µm 1.0 × 150 mm, (Waters) protected by a 0.2 µm in-line filter and maintained at 40 °C. The mobile phase consisted of 1% (*v*/*v*) formic acid in Milli-Q water (solvent A) and 1% (*v*/*v*) formic acid in methanol (solvent B). Elution was performed using a gradient system at a flow rate of 0.08 mL·min^−1^: 2%–30% B in 15 min; 30%–50% B in 15 min, 50%–60% B in 5 min, 60%–2% B in 6min and reequilibration (2% B for 5 min). Injection volume was 0.5 µL. Detection was operated at wavelength ranging from 250 to 600 nm. ESI conditions were as follows: HV capillary voltage, 2.5 kV; drying temperature, 200 °C; Nitrogen nebulizer gas, 10 psi; Nitrogen drying gas, 5 L·min^−1^; capillary exit voltage, 140 V; and syringe draw rate, 10 µL·min^−1^. The [M−H]^−^, [M+H]^+^ and [M+2H]^2+^ ions were selected for CID fragmentation (with helium as collision gas) to produce the MS/MS spectra.

These qualitative analyses were performed on the 12 selected rosé wines in order to establish a list of quantifiable polyphenols as extensive as possible, and potentially to detect new compounds. The MRM transitions were subsequently adapted to the UHPLC-QqQ-MS method.

### 3.5. UHPLC-QqQ-MS Conditions

UHPLC-QqQ-MS analyses were carried out using an Acquity UPLC system (Waters) hyphenated to a triple quadrupole mass spectrometer (Waters) with electrospray ionization (ESI) operating in switching positive and negative modes. The UHPLC system included a binary pump, a cooled autosampler maintained at 7 °C and equipped with a 5-µL sample loop, a 100-µL syringe and a 30-µL needle, and a DAD. MassLynx software was used to control the instruments and to acquire the data, and TargetLynx software was used to process the data. The column used for chromatographic separation was a reversed-phase Acquity HSS T3 1.8 µm 1.0 × 100 mm, (Waters) protected by a 0.2 µm in-line filter and maintained at 40 °C. The mobile phase consisted of 1% (*v*/*v*) formic acid in Milli-Q water (solvent A) and 1% (*v*/*v*) formic acid in methanol (solvent B). The source and desolvation temperatures were respectively set at 120 °C and 450 °C. Nitrogen was used as desolvation (500 L·h^−1^) and cone (50 L·h^−1^) gas. Argon was used as collision gas at a flow rate of 0.16 mL·min^−1^. Capillary voltage was set at 3.5 kV in positive mode and 2.8 kV in negative mode. The MRM transitions are described in Table 1.

Samples were injected into the column by using the Partial Loop with Needle Overfill injection mode, with an injection volume of 2 µL. The following gradient elution program, at a flow rate of 0.17 mL·min^−1^, was performed: isocratic 1% B from 0.0 to 2.0 min, linear 1%–5% B from 2.0 to 2.1 min, linear 5%–10% B from 2.1 to 8.0 min, linear 10%–28% B from 8.0 to 12.0 min, isocratic 28% B from 12.0 to 18.0 min, linear 28%–45% B from 18.0 to 22.0 min, linear 45%–99% B from 22.0 to 23.5 min, isocratic 99% B from 23.5 to 26.5 min. At the end of this sequence, the column was brought back to initial conditions with linear 99%–1% B from 26.5 to 27.0 min, then re-equilibrated with isocratic 1% B from 27.0 to 30.0 min. Two detectors were used: DAD scanning from 210 to 600 nm (resolution 1.2 nm) and the QqQ detector.

## 4. Conclusions

We have developed a high-throughput UHPLC-QqQ-MS analysis method for the quantification of 152 phenolic compounds, and two non-phenolic compounds in rosé wines in 30 min, without sample pre-treatment apart from filtering. Our method combines selectivity and sensitivity, both enhanced by the use of UHPLC and a QqQ-MRM acquisition mode alternating positive and negative ionization modes in a single run. The quantitative analysis of 12 rosé wines from different regions of the world showed the suitability of this method for large-scale wine analyses. Such a study is on its way and will aim to establish a typology of worldwide rosé wines by correlating chemometrically the phenolic composition to the colour, the geographic origin and the grape varieties. This quantification method can also be considered for metabolomics’ approaches as well as for fraud detection (*i.e.*, illegal wine blending).

## Supplementary Materials

Supplementary materials can be accessed at: http://www.mdpi.com/1420-3049/20/05/7890/s1.
