# Supplementary material for: A High-Throughput UHPLC-QqQ-MS Method for Polyphenol Profiling in Rosé Wines
Source: Molecules. 2015 Apr 30;20(5):7890–914. doi: 10.3390/molecules20057890 (PMC6272418; doi:10.3390/molecules20057890)
Supplement: Supplementary file 1 [file molecules-20-07890-s001.pdf]

Supplementary Materials

Table S1. Measured polyphenol concentration (Conc.) and calculated Limit Of Quantification (LOQ) in the 12 rosé wines (n.d.: not detected).

		**1**		**2**		**3**		**4**	
		**Conc. (mg·L^{-1})**	**LOQ (mg·L^{-1})**	**Conc. (mg·L^{-1})**	**LOQ (mg·L^{-1})**	**Conc. (mg·L^{-1})**	**LOQ (mg·L^{-1})**	**Conc. (mg·L^{-1})**	**LOQ (mg·L^{-1})**
Benzoic acids and derivatives	*Gallic acid*	10.53 ± 0.09	0.08	0.427 ± 0.009	0.2	1.34 ± 0.02	0.2	3.5 ± 0.1	0.08
	Protocatechuic acid	2.46 ± 0.02	0.07	0.54 ± 0.02	0.02	0.572 ± 0.006	0.03	1.51 ± 0.03	0.04
	Syringic acid	1.68 ± 0.06	0.07	1.11 ± 0.05	0.03	0.327 ± 0.008	0.03	0.63 ± 0.03	0.04
	Vanillic acid	1.5 ± 0.2	0.3	1.1 ± 0.3	0.3	0.37 ± 0.06	0.3	0.86 ± 0.05	0.2
	Ethyl gallate	0.9 ± 0.3	0.005	0.06 ± 0.03	0.05	0.18 ± 0.06	0.005	0.4 ± 0.2	0.007
	Ethyl protocatechuate	0.12 ± 0.1	<0.02	0.06 ± 0.05	<0.02	0.03 ± 0.02	0.02	0.21 ± 0.1	<0.02
Hydroxycinnamic acids and derivatives	*Caffeic acid*	2.88 ± 0.03	0.2	0.69 ± 0.02	0.07	3.4 ± 0.2	0.05	10.5 ± 0.8	0.06
	p-coumaric acid	1.3 ± 0.3	0.5	0.3 ± 0.2	0.5	0.8 ± 0.2	0.3	4.1 ± 0.7	0.3
	Ferulic acid	0.4 ± 0.2	0.05	0.1 ± 0.2	0.2	0.2 ± 0.2	0.2	0.9 ± 0.3	0.4
	Caftaric acid (cis- and trans- isomers)	40.6 ± 0.6	0.3	4.97 ± 0.1	0.5	53.6 ± 0.4	0.3	5.74 ± 0.06	0.4
	Coutaric acid (cis- and trans- isomers)	20.5 ± 0.6	0.02	2.9 ± 0.4	0.02	12.2 ± 0.5	0.06	3 ± 2	0.02
	Fertaric acid	4.4 ± 0.2	0.02	3.91 ± 0.08	0.01	9.7 ± 0.5	0.004	6.5 ± 0.4	0.003
	GRP (cis- and trans- isomers)	6.9 ± 0.3	0.2	10.1 ± 0.2	0.08	23 ± 3	0.06	10 ± 2	0.04
	Ethyl caffeate	0.14 ± 0.07	0.008	0.11 ± 0.02	0.009	1.4 ± 0.3	0.008	6.2 ± 0.3	0.07
	Ethyl coumarate	0.08 ± 0.08	0.01	0.014 ± 0.005	0.005	0.23 ± 0.09	0.007	0.67 ± 0.1	0.008
Stilbens	*trans-resveratrol*	0.133 ± 0.005	0.05	0.185 ± 0.002	0.02	0.085 ± 0.009	0.02	0.22 ± 0.04	0.04
	cis-resveratrol	0.059 ± 0.008	0.02	0.034 ± 0.005	0.02	0.046 ± 0.007	0.02	0.186 ± 0.009	0.03
	viniferin-1	0.24 ± 0.09	0.4	0.09 ± 0.04	0.3	0.08 ± 0.05	0.2	0.11 ± 0.05	0.2
	viniferin-2	0.01 ± 0.02	0.02	0.029 ± 0.002	0.05	0.018 ± 0.003	0.02	0.021 ± 0.005	0.04
	trans-piceid	1.41 ± 0.09	0.5	0.9 ± 0.2	0.7	0.23 ± 0.05	0.4	0.4 ± 0.04	0.3
	cis-piceid	0.59 ± 0.06	0.3	1.3 ± 0.2	0.09	0.57 ± 0.04	0.06	1.25 ± 0.04	0.3
Flavonols	*Quercetin Glc*	0.017 ± 0.003	0.03	0.013 ± 0.002	0.02	0.008 ± 0.005	0.02	0.004 ± 0.004	0.01
	Myricetin glucuronide	0.12 ± 0.04	2	0.004 ± 0.002	0.2	0.003 ± 0.003	0.2	0.021 ± 0.006	0.6
	Myricetin Glc	0.19 ± 0.02	0.007	0.012 ± 0.003	0.004	0.01 ± 0.002	0.002	0.051 ± 0.007	0.004
	Quercetin glucuronide	0.44 ± 0.03	0.006	0.12 ± 0.03	0.002	0.26 ± 0.07	0.004	0.31 ± 0.03	0.005
	Quercetin	n.d.	0.2	0.01 ± 0.02	0.2	0.01 ± 0.02	0.02	0.005 ± 0.01	0.03

Table S1. *Cont.*

		1		2		3		4	
		**Conc. (mg·L^{-1})**	**LOQ (mg·L^{-1})**	**Conc. (mg·L^{-1})**	**LOQ (mg·L^{-1})**	**Conc. (mg·L^{-1})**	**LOQ (mg·L^{-1})**	**Conc. (mg·L^{-1})**	**LOQ (mg·L^{-1})**
Flavan-3-ols	*Catechin*	19.9 ± 0.2	0.3	2.8 ± 0.4	0.08	4.6 ± 0.3	0.04	3.177 ± 0.241	0.08
	Galloylated dimer	n.d.	<0.002	n.d.	<0.002	n.d.	<0.002	n.d.	<0.002
	Trimer-1 (1 isomer)	0.44 ± 0.05	0.02	0.02 ± 0.02	<0.007	0.08 ± 0.03	<0.007	0.041 ± 0.008	0.007
	Trimers-2 (several isomers)	0.209 ± 0.004	<0.002	0.001 ± 0.003	0.03	0.024 ± 0.002	0.03	0.035 ± 0.005	0.03
	(epi)cat-ethyl-(epi)cat-1 (1 isomer)	n.d.	<0.08	n.d.	<0.08	n.d.	<0.08	n.d.	<0.08
	(epi)cat-ethyl-(epi)cat-2 (2 isomers)	n.d.	<0.0007	n.d.	0.008	n.d.	0.02	n.d.	0.001
	Epicatechin	5.5 ± 0.3	3	1.8 ± 0.2	0.6	0.8 ± 0.6	0.2	1.9 ± 0.7	0.9
	Dimer B2	2.73 ± 0.07	0.06	0.48 ± 0.02	0.05	0.33 ± 0.02	0.04	0.94 ± 0.03	0.03
	Dimer B1	18.7 ± 0.2	0.05	1.96 ± 0.05	0.05	5.3 ± 0.2	0.03	2.9 ± 0.2	0.03
	Dimer B3	1.04 ± 0.05	0.04	0.165 ± 0.007	0.02	0.17 ± 0.02	0.02	0.218 ± 0.008	0.04
	Dimer B4	0.25 ± 0.08	0.4	0.06 ± 0.04	0.06	n.d.	0.2	0.19 ± 0.06	0.07
Dihydroflavonols	*Astilbin*	0.84 ± 0.04	0.003	0.75 ± 0.02	0.002	0.488 ± 0.009	0.001	0.98 ± 0.03	0.01
	Taxifolin	0.73 ± 0.03	0.2	0.32 ± 0.02	0.2	0.25 ± 0.05	0.07	0.46 ± 0.04	0.2
Anthocyanins	*Malvidin 3,5-diGlc*	0.045 ± 0.004	0.4	0.38 ± 0.02	0.2	0.03 ± 0.02	0.08	0.067 ± 0.007	0.6
	Delphinidin 3,5-diGlc	0.052 ± 0.014	0.02	0.036 ± 0.01	0.02	n.d.	0.02	0.042 ± 0.006	0.02
	Cyanidin 3,5-diGlc	0.021 ± 0.004	0.02	n.d.	0.02	n.d.	0.02	0.01 ± 0.02	0.02
	Petunidin 3,5-diGlc	n.d.	0.02	n.d.	0.02	n.d.	0.02	n.d.	0.02
	Peonidin 3,5-diGlc	0.04 ± 0.02	0.2	0.21 ± 0.03	0.1	0.005 ± 0.009	0.02	0.036 ± 0.005	0.2
	Malvidin 3-O-Glc	6.68 ± 0.03	0.02	2.88 ± 0.05	0.2	1.6 ± 0.02	0.005	4.38 ± 0.1	0.04
	Delphinidin 3-O-Glc	0.316 ± 0.006	0.03	0.0167 ± 0.0008	0.02	0.0142 ± 0.0004	0.002	0.16 ± 0.02	0.01
	Cyanidin 3-O-Glc	0.0465 ± 0.0006	0.002	0.0065 ± 0.0005	0.004	0.005 ± 0.002	0.0007	0.021 ± 0.002	0.002
	Petunidin 3-O-Glc	0.8 ± 0.03	0.02	0.036 ± 0.005	0.002	0.07 ± 0.006	0.005	0.41 ± 0.02	0.02
	Peonidin 3-O-Glc	1.25 ± 0.02	0.03	0.9 ± 0.03	0.06	0.203 ± 0.006	0.004	0.56 ± 0.01	0.02
	Delphinidin 3-O-acetyl-Glc	0.0269 ± 0.0004	0.003	0.0027 ± 0.0009	0.002	0.0044 ± 0.0003	0.002	0.086 ± 0.004	0.003
	Cyanidin 3-O-acetyl-Glc	0.0086 ± 0.0004	0.002	0.0009 ± 0.0002	0.0007	0.0068 ± 0.0003	0.0007	0.036 ± 0.003	0.002
	Petunidin 3-O-acetyl-Glc	0.046 ± 0.002	0.002	0.0031 ± 0.0004	0.002	0.0128 ± 0.001	0.002	0.132 ± 0.006	0.0009
	Peonidin 3-O-acetyl-Glc	0.131 ± 0.006	0.0008	0.016 ± 0.002	0.0003	0.161 ± 0.005	0.0005	0.45 ± 0.02	0.002

Table S1. *Cont.*

		1		2		3		4	
		**Conc. (mg·L⁻¹)**	**LOQ (mg·L⁻¹)**	**Conc. (mg·L⁻¹)**	**LOQ (mg·L⁻¹)**	**Conc. (mg·L⁻¹)**	**LOQ (mg·L⁻¹)**	**Conc. (mg·L⁻¹)**	**LOQ (mg·L⁻¹)**
Anthocyanins	*Malvidin 3-O-acetyl-Glc*	0.565 ± 0.005	0.003	0.049 ± 0.002	0.0008	0.451 ± 0.003	0.004	1.73 ± 0.06	0.002
	Delphinidin 3-O-coumaroyl-Glc (cis- and trans- isomers)	0.05 ± 0.02	0.002	0.001 ± 0.002	0.004	0 ± 0.0001	0.002	0.018 ± 0.005	0.0006
	Cyanidin 3-O-coumaroyl-Glc (cis- and trans- isomers)	0.032 ± 0.006	0.004	0.002 ± 0.003	0.0009	0.008 ± 0.003	0.002	0.007 ± 0.004	0.0004
	Petunidin 3-O-coumaroyl-Glc (cis- and trans- isomers)	0.095 ± 0.006	0.005	0.005 ± 0.002	0.002	0.011 ± 0.002	0.0005	0.023 ± 0.005	0.002
	Peonidin 3-O-coumaroyl-Glc (cis- and trans- isomers)	0.271 ± 0.006	0.003	0.0113 ± 0.0006	0.0002	0.09 ± 0.002	0.002	0.076 ± 0.002	0.002
	Malvidin 3-O-coumaroyl-Glc (cis- and trans- isomers)	0.93 ± 0.03	0.004	0.076 ± 0.002	0.0004	0.213 ± 0.003	0.0009	0.282 ± 0.008	0.003
	Delphinidin 3-O-caffeoyl-Glc	n.d.	0.0009	n.d.	0.002	n.d.	0.003	n.d.	0.03
	Cyanidin 3-O-caffeoyl-Glc	n.d.	0.0005	n.d.	0.0005	n.d.	0.0006	n.d.	0.002
	Petunidin 3-O-caffeoyl-Glc	n.d.	0.0006	n.d.	0.0009	n.d.	0.0005	n.d.	0.0006
	Peonidin 3-O-caffeoyl-Glc	0.006 ± 0.005	0.0007	n.d.	0.0003	0.002 ± 0.002	0.0006	0.002 ± 0.003	0.0008
	Malvidin 3-O-caffeoyl-Glc	0.012 ± 0.004	0.002	0.006 ± 0.008	0.0008	0.005 ± 0.007	0.0009	0.003 ± 0.002	0.002
	(epi)cat-ethyl-peonidin 3-O-Glc-1	n.d.	0.002	n.d.	0.0007	n.d.	0.0008	n.d.	0.003
	(epi)cat-ethyl-peonidin 3-O-Glc-2	n.d.	0.002	n.d.	0.0007	n.d.	0.0008	n.d.	0.003
	(epi)cat-ethyl-peonidin 3-O-Glc-3	0.0005 ± 0.0009	0.002	n.d.	0.0007	n.d.	0.0008	n.d.	0.003
	(epi)cat-ethyl-peonidin 3-O-Glc-4	n.d.	0.002	n.d.	0.0007	n.d.	0.0008	n.d.	0.003
	(epi)cat-ethyl-malvidin 3-O-Glc-1	n.d.	0.002	n.d.	0.003	n.d.	0.003	n.d.	0.003
	(epi)cat-ethyl-malvidin 3-O-Glc-2	n.d.	0.002	n.d.	0.003	n.d.	0.003	n.d.	0.003
	(epi)cat-ethyl-malvidin 3-O-Glc-3 and 4	0.001 ± 0.002	0.002	n.d.	0.003	n.d.	0.003	n.d.	0.003
	(epi)cat-ethyl-malvidin 3-O-coumaroyl-Glc (2 co-eluted isomers)	0.005 ± 0.008	0.003	n.d.	0.0004	0.002 ± 0.004	0.0004	n.d.	0.0006
	Delphinidin 3-O-Glc-(epi)cat	n.d.	0.002	n.d.	0.003	n.d.	0.003	n.d.	0.003
	Cyanidin 3-O-Glc-(epi)cat	0.003 ± 0.003	0.04	n.d.	0.002	n.d.	0.004	n.d.	0.002
	Petunidin 3-O-Glc-(epi)cat	0.015 ± 0.008	0.06	n.d.	0.003	n.d.	0.003	n.d.	0.005

Table S1. *Cont.*

		1		2		3		4	
		**Conc. (mg·L^{-1})**	**LOQ (mg·L^{-1})**	**Conc. (mg·L^{-1})**	**LOQ (mg·L^{-1})**	**Conc. (mg·L^{-1})**	**LOQ (mg·L^{-1})**	**Conc. (mg·L^{-1})**	**LOQ (mg·L^{-1})**
Anthocyanins	*Peonidin 3-O-Glc-(epi)cat*	0.07 ± 0.04	0.002	0.005 ± 0.006	0.002	0.0004 ± 0.0008	0.002	0.02 ± 0.02	0.003
	Malvidin 3-O-Glc-(epi)cat (2 isomers)	0.16 ± 0.009	0.005	0.014 ± 0.001	0.002	0.007 ± 0.002	0.001	0.044 ± 0.004	0.02
	Malvidin 3-O-coumaroyl-Glc-(epi)cat (2 isomers)	0.0004 ± 0.0004	0.005	n.d.	0.003	n.d.	0.0008	n.d.	0.002
	(epi)cat-delphinidin 3-O-Glc A-F bicyclic	0.001 ± 0.003	0.002	n.d.	0.002	n.d.	0.005	n.d.	0.007
	(epi)cat-cyanidin 3-O-Glc A-F bicyclic	n.d.	0.002	n.d.	0.003	n.d.	0.002	n.d.	0.004
	(epi)cat-petunidin 3-O-Glc A-F bicyclic	0.006 ± 0.009	0.005	n.d.	0.004	n.d.	0.005	n.d.	0.006
	(epi)cat-peonidin 3-O-Glc A-F bicyclic	0.004 ± 0.004	0.002	n.d.	0.007	n.d.	0.006	0.002 ± 0.004	0.003
	(epi)cat-malvidin 3-O-Glc A-F bicyclic	0.005 ± 0.004	0.04	n.d.	0.002	0.0004 ± 0.0008	0.002	0.0005 ± 0.0009	0.002
	Caftaric-peonidin 3-O-Glc (2 isomers)	0.005 ± 0.006	0.002	0.03 ± 0.02	0.002	0.0003 ± 0.0002	0.002	0.003 ± 0.003	0.002
	Caftaric-malvidin 3-O-Glc (2 isomers)	0.026 ± 0.008	0.002	0.04 ± 0.008	0.002	0.009 ± 0.002	0.001	0.006 ± 0.006	0.001
	Coutaric-malvidin 3-O-Glc (2 isomers)	n.d.	0.02	n.d.	0.002	n.d.	0.002	n.d.	0.002
	Pyranodelphinidin 3-O-Glc	0.001 ± 0.002	0.03	n.d.	0.006	n.d.	0.0009	n.d.	0.002
	Pyranocyanidin 3-O-Glc	0.0004 ± 0.0007	0.02	n.d.	0.02	n.d.	0.06	n.d.	0.03
	Pyranopetunidin 3-O-Glc	0.0001 ± 0.0003	0.008	n.d.	0.03	0 ± 0.0001	0.02	0.001 ± 0.002	0.02
	Pyranopeonidin 3-O-Glc	0.003 ± 0.002	0.003	0.0008 ± 0.0009	0.003	0.0004 ± 0.0003	0.003	0.0003 ± 0.0006	0.005
	Pyranomalvidin 3-O-Glc (vitisin B)	0.014 ± 0.003	0.007	0.0009 ± 0.0009	0.005	0.0021 ± 0.0004	0.002	0.006 ± 0.002	0.005
	Carboxypyranodelphinidin 3-O-Glc	0.01 ± 0.01	0.02	0.0002 ± 0.0004	0.003	n.d.	0.002	0.004 ± 0.002	0.005
	Carboxypyranocyanidin 3-O-Glc	n.d.	0.02	n.d.	0.0003	n.d.	0.009	n.d.	0.03
	Carboxypyranopetunidin 3-O-Glc	0.005 ± 0.006	0.0008	n.d.	0.03	n.d.	0.009	0.001 ± 0.002	0.02
	Carboxypyranopeonidin 3-O-Glc	0.027 ± 0.008	0.0004	0.0034 ± 0.001	0.0004	0.0013 ± 0.0008	0.0004	0.007 ± 0.003	0.0005
	Carboxypyranomalvidin 3-O-Glc (vitisin A)	0.12 ± 0.02	0.003	0.012 ± 0.003	0.002	0.0114 ± 0.0009	0.0004	0.086 ± 0.01	0.005
	Pyranodelphinidin 3-O-acetyl-Glc	n.d.	0.003	n.d.	0.0006	n.d.	0.0006	n.d.	0.005
	Pyranocyanidin 3-O-acetyl-Glc	0.0003 ± 0.0003	0.008	0.0001 ± 0.0002	0.0007	n.d.	0.0009	0.0001 ± 0.0002	0.002
	Pyranopetunidin 3-O-acetyl-Glc	n.d.	0.0002	n.d.	0.0005	n.d.	0.0005	n.d.	0.0007
	Pyranopeonidin 3-O-acetyl-Glc	n.d.	0.001	n.d.	0.001	n.d.	0.0006	n.d.	0.0003
	Pyranomalvidin 3-O-acetyl-Glc	n.d.	0.0004	n.d.	0.0005	n.d.	0.002	n.d.	0.0004
	Carboxypyranodelphinidin 3-O-acetyl-Glc	n.d.	0.001	n.d.	0.0005	n.d.	0.0008	n.d.	0.0008

Table S1. *Cont.*

		1		2		3		4	
		**Conc. (mg·L^{-1})**	**LOQ (mg·L^{-1})**	**Conc. (mg·L^{-1})**	**LOQ (mg·L^{-1})**	**Conc. (mg·L^{-1})**	**LOQ (mg·L^{-1})**	**Conc. (mg·L^{-1})**	**LOQ (mg·L^{-1})**
Anthocyanins	*Carboxypyranocyanidin 3-O-acetyl-Glc*	0.0002 ± 0.0004	0.002	n.d.	0.01	n.d.	0.0003	0.0002 ± 0.0004	0.0006
	Carboxypyranopetunidin 3-O-acetyl-Glc	n.d.	0.003	n.d.	0.0004	n.d.	0.0006	0.0002 ± 0.0003	0.0004
	Carboxypyranopeonidin 3-O-acetyl-Glc	n.d.	0.005	n.d.	0.002	n.d.	0.04	0.0008 ± 0.0007	0.003
	Carboxypyranomalvidin 3-O-acetyl-Glc	0.008 ± 0.004	0.001	n.d.	0.002	0.002 ± 0.004	0.002	0.017 ± 0.003	0.002
	Pyranopetunidin 3-O-coumaroyl-Glc	n.d.	0.0007	n.d.	0.0003	n.d.	0.0004	n.d.	0.0005
	Pyranopeonidin 3-O-coumaroyl-Glc	n.d.	0.008	n.d.	0.0008	n.d.	0.003	n.d.	0.002
	Pyranomalvidin 3-O-coumaroyl-Glc	0.001 ± 0.003	0.0007	n.d.	0.002	n.d.	0.003	n.d.	0.0009
	Carboxypyranopetunidin 3-O-coumaroyl-Glc	0.001 ± 0.002	0.002	n.d.	0.002	n.d.	0.002	n.d.	0.002
	Carboxypyranopeonidin 3-O-coumaroyl-Glc	0.004 ± 0.004	0.0005	n.d.	0.0005	n.d.	0.0004	0.001 ± 0.002	0.002
	Carboxypyranomalvidin 3-O-coumaroyl-Glc	0.008 ± 0.002	0.0004	n.d.	0.0004	n.d.	0.0004	0.002 ± 0.004	0.0002
	p-hydroxyphenylpyranopeonidin 3-O-Glc	0.006 ± 0.003	0.004	0.011 ± 0.002	0.02	0.006 ± 0.005	0.004	0.05 ± 0.02	0.05
	p-hydroxyphenylpyranomalvidin 3-O-Glc	0.04 ± 0.02	0.0005	0.05 ± 0.02	0.0003	0.055 ± 0.005	0.0002	0.49 ± 0.03	0.0003
	p-hydroxyphenylpyranopeonidin 3-O-acetyl-Glc	0.0003 ± 0.0006	0.0004	n.d.	0.0005	0.005 ± 0.002	0.0003	0.027 ± 0.008	0.0002
	p-hydroxyphenylpyranomalvidin 3-O-acetyl-Glc	0.0026 ± 0.0004	0. 0	0.001 ± 0.002	0.0002	0.012 ± 0.005	0.0002	0.13 ± 0.04	0.0002
	p-hydroxyphenylpyranopeonidin 3-O-coumaroyl-Glc	0.0006 ± 0.0003	0.0004	n.d.	0.0002	0.001 ± 0.002	0.0004	0.0191 ± 0.0004	0.0004
	p-hydroxyphenylpyranomalvidin 3-O-coumaroyl-Glc	0.007 ± 0.002	0.0006	0.0004 ± 0.0007	0.0004	0.018 ± 0.009	0.0005	0.08 ± 0.02	0.0002
	Catechylpyranopeonidin 3-O-Glc	0.013 ± 0.003	0.0003	0.001 ± 0.002	0.0002	0.002 ± 0.002	0.0004	0.06 ± 0.02	0.0004
	Catechylpyranomalvidin 3-O-Glc (pinotin A)	0.07 ± 0.02	0.005	0.003 ± 0.002	0.0006	0.026 ± 0.009	0.0005	0.108 ± 0.01	0.0003
	Catechylpyranopetunidin 3-O-acetyl-Glc	0.0004 ± 0.0007	0.0002	n.d.	0.0007	n.d.	0.0002	0.013 ± 0.007	0.0002
	Catechylpyranopeonidin 3-O-acetyl-Glc	n.d.	0.0002	n.d.	0.0007	0.0002 ± 0.0003	0.0003	0.015 ± 0.007	0.0004
	Catechylpyranomalvidin 3-O-acetyl-Glc	0.002 ± 0.003	0.0005	0.0001 ± 0.0002	0.0005	0.005 ± 0.003	0.0005	0.054 ± 0.007	0.0006
	Catechylpyranopetunidin 3-O-coumaroyl-Glc	n.d.	0.0004	n.d.	0.0004	0.0001 ± 0.0002	0.0002	0.005 ± 0.005	0.0002
	Catechylpyranopeonidin 3-O-coumaroyl-Glc	0.003 ± 0.002	0.0002	n.d.	0.0002	0.003 ± 0.003	0.0003	0.015 ± 0.006	0.00
	Catechylpyranomalvidin 3-O-coumaroyl-Glc	0.018 ± 0.008	0.0005	n.d.	0.0002	0.004 ± 0.003	0.0003	0.019 ± 0.006	0.003
	Guaiacylpyranomalvidin 3-O-Glc	0.01 ± 0.009	0.0006	0.049 ± 0.01	0.0002	0.059 ± 0.016	0.0002	0.19 ± 0.03	0.0003
	Guaiacylpyranomalvidin 3-O-acetyl-Glc	0.0002 ± 0.0003	0.0002	0.001 ± 0.002	0.0002	0.0096 ± 0.0009	0.0003	0.057 ± 0.009	0.0003

Table S1. *Cont.*

		1		2		3		4	
		**Conc. (mg·L^{-1})**	**LOQ (mg·L^{-1})**	**Conc. (mg·L^{-1})**	**LOQ (mg·L^{-1})**	**Conc. (mg·L^{-1})**	**LOQ (mg·L^{-1})**	**Conc. (mg·L^{-1})**	**LOQ (mg·L^{-1})**
Anthocyanins	*Guaiacylpyranomalvidin 3-O-coumaroyl-Glc*	0.003 ± 0.002	0.0003	0.0001 ± 0.0003	0.0009	0.016 ± 0.004	0.0003	0.022 ± 0.004	0.002
	Syringylpyranomalvidin 3-O-Glc	n.d.	0.007	n.d.	0.002	n.d.	0.0009	0 ± 0.0001	0.002
	Petunidin 3-O-acetyl-Glc-(epi)cat	n.d.	0.003	n.d.	0.003	n.d.	0.002	n.d.	0.04
	Peonidin 3-O-acetyl-Glc-(epi)cat	n.d.	0.002	n.d.	0.005	n.d.	0.003	n.d.	0.002
	Malvidin 3-O-acetyl-Glc-(epi)cat	n.d.	0.002	n.d.	0.002	n.d.	0.0004	n.d.	0.001
	Pyranopeonidin 3-O-Glc-(epi)cat	0.0021 ± 0.0004	0.0005	n.d.	0.0010	n.d.	0.0007	0.0008 ± 0.0008	0.0005
	Pyranomalvidin 3-O-Glc-(epi)cat	0.004 ± 0.004	0.0004	n.d.	0.0003	n.d.	0.0002	0.001 ± 0.002	0.0003
	Pyranomalvidin 3-O-coumaroyl-Glc-(epi)cat	n.d.	0.002	n.d.	0.002	n.d.	0.002	n.d.	0.002
	Unknown "551"	0.014 ± 0.01	0.02	0.003 ± 0.003	0.0006	n.d.	0.003	0.002 ± 0.004	0.002
	Unknown "581"	0.032 ± 0.01	0.004	0.004 ± 0.002	0.002	0.001 ± 0.002	0.003	0.021 ± 0.006	0.004
	Unknown "1095"	n.d.	0.02	n.d.	0.02	n.d.	0.03	n.d.	0.02
	Unknown "1099"	n.d.	0.02	n.d.	0.004	n.d.	0.002	0.001 ± 0.0003	0.007
Others (aminoacids and alcohols)	*Tyrosine*	12.68 ± 0.02	0.4	8.62 ± 0.06	0.6	8.15 ± 0.07	0.7	7.81 ± 0.074	0.7
	Tyrosol	7.97 ± 0.139	4	6.585 ± 0.143	2	9.9 ± 0.3	2	10.3 ± 0.6	4
	Hydroxytyrosol	0.4 ± 0.04	0.07	0.08 ± 0.02	0.2	0.12 ± 0.02	0.03	0.77 ± 0.03	0.2
	Tryptophol	0.2 ± 0.1	0.2	0.17 ± 0.03	0.3	0.4 ± 0.2	0.2	0.5 ± 0.3	0.2
	Tryptophan	1.2 ± 0.2	0	1.6 ± 0.5	0	0.8 ± 0.3	0	1 ± 0.2	0

Table S1. *Cont.*

		5		6		7		8	
		**Conc. (mg·L^{-1})**	**LOQ (mg·L^{-1})**	**Conc. (mg·L^{-1})**	**LOQ (mg·L^{-1})**	**Conc. (mg·L^{-1})**	**LOQ (mg·L^{-1})**	**Conc. (mg·L^{-1})**	**LOQ (mg·L^{-1})**
Benzoic acids and derivatives	*Gallic acid*	1.71 ± 0.06	0.1	13.8 ± 0.4	0.07	1.48 ± 0.04	0.04	2.36 ± 0.06	0.08
	Protocatechuic acid	1.27 ± 0.05	0.06	2.2 ± 0.02	0.07	0.67 ± 0.02	0.02	1.4 ± 0.2	0.05
	Syringic acid	2.7 ± 0.6	0.05	2.07 ± 0.03	0.05	0.48 ± 0.02	0.08	0.55 ± 0.003	0.04
	Vanillic acid	1.72 ± 0.1	0.2	1.55 ± 0.03	0.2	0.3 ± 0.2	0.05	0.86 ± 0.06	0.05
	Ethyl gallate	0.25 ± 0.1	0.006	2 ± 0.3	0.006	0.11 ± 0.03	0.01	0.37 ± 0.03	0.005
	Ethyl protocatechuate	0.3 ± 0.09	<0.02	0.7 ± 0.2	<0.02	0.07 ± 0.04	<0.02	0.3 ± 0.2	<0.02
Hydroxycinnamic acids and derivatives	*Caffeic acid*	2.33 ± 0.07	0.09	3.4 ± 0.2	0.06	1.69 ± 0.04	0.2	2.2 ± 0.4	0.06
	p-coumaric acid	1.6 ± 0.2	0.3	1.3 ± 0.1	0.5	1.21 ± 0.08	0.4	0.7 ± 0.2	0.3
	Ferulic acid	0.93 ± 0.07	0.02	0.11 ± 0.08	0.06	0.5 ± 0.2	0.02	0.2 ± 0.2	0.04
	Caftaric acid (cis- and trans- isomers)	30.9 ± 0.7	0.3	23.8 ± 0.4	0.3	26.3 ± 0.4	0.5	48 ± 3	0.2
	Coutaric acid (cis- and trans- isomers)	10 ± 2	0.03	7.705 ± 0.783	0.02	12.5 ± 0.3	0.07	13 ± 0.9	0.03
	Fertaric acid	6.3 ± 0.2	0.006	3.75 ± 0.1	0.008	5.23 ± 0.06	0.007	6.8 ± 0.3	0.02
	GRP (cis- and trans- isomers)	9 ± 2	0.2	0.44 ± 0.08	0.4	15.5 ± 0.3	0.2	11 ± 2	0.2
	Ethyl caffeate	0.5 ± 0.2	0.006	0.22 ± 0.05	0.03	0.5 ± 0.2	0.04	0.39 ± 0.08	0.008
	Ethyl coumarate	0.15 ± 0.07	0.02	0.087 ± 0.009	0.02	0.3 ± 0.2	0.01	0.14 ± 0.04	0.05
Stilbens	*trans-resveratrol*	0.57 ± 0.04	0.04	0.264 ± 0.008	0.02	0.15 ± 0.009	0.03	0.16 ± 0.02	0.02
	cis-resveratrol	0.53 ± 0.04	0.04	0.416 ± 0.005	0.03	0.049 ± 0.007	0.04	0.11 ± 0.02	0.04
	viniferin-1	0.17 ± 0.09	0. 3	0.22 ± 0.03	0.3	0.098 ± 0.007	0.2	0.042 ± 0.008	0.07
	viniferin-2	0.029 ± 0.007	0.04	n.d.	0.02	n.d.	0.03	0.011 ± 0.01	0.03
	trans-piceid	0.93 ± 0.03	0.3	0.38 ± 0.03	0.8	0.43 ± 0.07	0.7	0.4 ± 0.2	0.8
	cis-piceid	2 ± 0.2	0.4	1.4 ± 0.2	0.06	1.04 ± 0.05	0. 2	1 ± 0.2	0.2
Flavonols	*Quercetin Glc*	0.009 ± 0.006	0.02	0.02 ± 0.005	0.009	0.01 ± 0.009	0.008	0.013 ± 0.003	0.02
	Myricetin glucuronide	0.028 ± 0.004	0.6	0.12 ± 0.05	4	0.016 ± 0.006	0.8	0.049 ± 0.003	0.5
	Myricetin Glc	0.052 ± 0.008	0.007	0.33 ± 0.07	0.0008	0.071 ± 0.009	0.002	0.044 ± 0.009	0.006
	Quercetin glucuronide	0.443 ± 0.006	0.007	1.39 ± 0.05	0.003	0.84 ± 0.04	0.003	0.68 ± 0.04	0.009
	Quercetin	n.d.	0.04	n.d.	0.02	n.d.	0.07	0.1 ± 0.05	0.03

Table S1. *Cont.*

		5		6		7		8	
		**Conc. (mg·L^{-1})**	**LOQ (mg·L^{-1})**	**Conc. (mg·L^{-1})**	**LOQ (mg·L^{-1})**	**Conc. (mg·L^{-1})**	**LOQ (mg·L^{-1})**	**Conc. (mg·L^{-1})**	**LOQ (mg·L^{-1})**
Flavan-3-ols	*Catechin*	6.3 ± 1	0.09	10.8 ± 0.2	0.3	5.2 ± 0.8	0.08	4.7 ± 0.5	0.06
	Galloylated dimer	n.d.	<0.002	n.d.	0.002	n.d.	<0.002	n.d.	<0.002
	Trimer-1 (1 isomer)	0.17 ± 0.03	<0.007	0.46 ± 0.06	<0.007	0.14 ± 0.04	<0.007	0.106 ± 0.007	<0.007
	Trimers-2 (several isomers)	0.07 ± 0.02	0.002	0.64 ± 0.04	0.06	0.07 ± 0.002	<0.002	0.024 ± 0.01	0.04
	(epi)cat-ethyl-(epi)cat-1 (1 isomer)	n.d.	0.2	n.d.	< 0.08	n.d.	<0.08	n.d.	<0.08
	(epi)cat-ethyl-(epi)cat-2 (2 isomers)	n.d.	0.003	n.d.	<0.0007	n.d.	<0.0007	n.d.	0.005
	Epicatechin	2.6 ± 0.2	1	6.3 ± 0.7	4	2.8 ± 1	0.7	3 ± 2	1
	Dimer B2	1.12 ± 0.03	0.04	5.04 ± 0.02	0.04	0.8 ± 0.05	0.06	1.22 ± 0.03	0.03
	Dimer B1	6.05 ± 0.05	0.04	12.6 ± 0.2	0.04	9.5 ± 0.2	0.04	5.9 ± 0.3	0.03
	Dimer B3	0.41 ± 0.02	0.02	1.59 ± 0.04	0.8	0.2 ± 0.02	0.04	0.29 ± 0.03	0.04
	Dimer B4	0.4 ± 0.2	0.08	1.5 ± 0.4	0.7	0.05 ± 0.03	0.4	0.12 ± 0.08	0.2
Dihydroflavonols	*Astilbin*	2.89 ± 0.04	0.03	0.2 ± 0.05	0.008	0.98 ± 0.02	0.002	0.1 ± 0.03	0.002
	Taxifolin	0.98 ± 0.03	0.3	0.18 ± 0.08	0.05	0.41 ± 0.05	0.3	0.19 ± 0.02	0.2
Anthocyanins	*Malvidin 3,5-diGlc*	0.23 ± 0.03	0.6	0.14 ± 0.03	2	0.053 ± 0.004	0.1	0.047 ± 0.008	0.6
	Delphinidin 3,5-diGlc	0.04 ± 0.02	0.02	0.063 ± 0.014	0.02	n.d.	0.02	0.06 ± 0.02	0.02
	Cyanidin 3,5-diGlc	n.d.	0.02	0.0191 ± 0.0009	0.02	n.d.	0.02	0.0226 ± 0.0005	0.02
	Petunidin 3,5-diGlc	n.d.	0.02	0.01 ± 0.02	0.03	n.d.	0.02	0.01 ± 0.02	0.05
	Peonidin 3,5-diGlc	0.112 ± 0.006	0.4	0.05 ± 0.02	0.2	0.028 ± 0.005	0.04	0.032 ± 0.002	0.2
	Malvidin 3-O-Glc	13.09 ± 0.1	0.05	17.3 ± 0.2	0.07	4.49 ± 0.1	0.02	11.6 ± 0.2	0.04
	Delphinidin 3-O-Glc	0.09 ± 0.006	0.02	0.47 ± 0.04	0.03	0.075 ± 0.003	0.002	0.432 ± 0.008	0.03
	Cyanidin 3-O-Glc	0.022 ± 0.001	0.002	0.052 ± 0.001	0.002	0.015 ± 0.002	0.005	0.06 ± 0.002	0.003
	Petunidin 3-O-Glc	0.42 ± 0.02	0.007	1.4 ± 0.2	0.02	0.28 ± 0.008	0.004	1.24 ± 0.03	0.02
	Peonidin 3-O-Glc	1.34 ± 0.02	0.04	0.967 ± 0.008	0.02	0.544 ± 0.004	0.008	1.28 ± 0.02	0.03
	Delphinidin 3-O-acetyl-Glc	0.01 ± 0.002	0.003	0.387 ± 0.004	0.004	0.0167 ± 0.0004	0.002	0.141 ± 0.003	0.0005
	Cyanidin 3-O-acetyl-Glc	0.0046 ± 0.0003	0.002	0.143 ± 0.003	0.004	0.0158 ± 0.0002	0.001	0.0771 ± 0.0002	0.002
	Petunidin 3-O-acetyl-Glc	0.0184 ± 0.0007	0.0006	0.593 ± 0.002	0.002	0.045 ± 0.002	0.003	0.245 ± 0.004	0.004
	Peonidin 3-O-acetyl-Glc	0.158 ± 0.003	0.001	1.71 ± 0.03	0.006	0.3497 ± 0.0007	0.002	0.911 ± 0.006	0.003

Table S1. *Cont.*

		5		6		7		8	
		**Conc. (mg·L^{-1})**	**LOQ (mg·L^{-1})**	**Conc. (mg·L^{-1})**	**LOQ (mg·L^{-1})**	**Conc. (mg·L^{-1})**	**LOQ (mg·L^{-1})**	**Conc. (mg·L^{-1})**	**LOQ (mg·L^{-1})**
Anthocyanins	*Malvidin 3-O-acetyl-Glc*	0.55 ± 0.02	0.003	11.509 ± 0.101	0.04	1.48 ± 0.02	0.004	3.259 ± 0.005	0.01
	Delphinidin 3-O-coumaroyl-Glc (cis- and trans- isomers)	0.01 ± 0.02	0.001	0.12 ± 0.04	0.0009	0.03 ± 0.02	0.0006	0.04 ± 0.02	0.02
	Cyanidin 3-O-coumaroyl-Glc (cis- and trans- isomers)	0.011 ± 0.006	0.0006	0.08 ± 0.02	0.003	0.022 ± 0.007	0.002	0.046 ± 0.005	0.0005
	Petunidin 3-O-coumaroyl-Glc (cis- and trans- isomers)	0.033 ± 0.004	0.0004	0.16 ± 0.03	0.007	0.0568 ± 0.0007	0.002	0.1 ± 0.02	0.005
	Peonidin 3-O-coumaroyl-Glc (cis- and trans- isomers)	0.21 ± 0.06	0.002	0.5 ± 0.2	0.006	0.214 ± 0.003	0.0009	0.34 ± 0.08	0.002
	Malvidin 3-O-coumaroyl-Glc (cis- and trans- isomers)	0.81 ± 0.02	0.002	2.5 ± 0.04	0.03	0.93 ± 0.02	0.002	1.15 ± 0.02	0.004
	Delphinidin 3-O-caffeoyl-Glc	n.d.	0.003	n.d.	0.002	n.d.	0.004	n.d.	0.003
	Cyanidin 3-O-caffeoyl-Glc	n.d.	0.0004	0.0006 ± 0.0002	0.002	n.d.	0.0008	n.d.	0.0008
	Petunidin 3-O-caffeoyl-Glc	n.d.	0.0007	n.d.	0.003	n.d.	0.002	n.d.	0.002
	Peonidin 3-O-caffeoyl-Glc	0.007 ± 0.007	0.004	0.01 ± 0.02	0.002	0.005 ± 0.005	0.0007	0.014 ± 0.008	0.0008
	Malvidin 3-O-caffeoyl-Glc	0.011 ± 0.006	0.0005	0.03 ± 0.02	0.02	0.026 ± 0.005	0.0007	0.025 ± 0.009	0.0008
	(epi)cat-ethyl-peonidin 3-O-Glc-1	n.d.	0.002	n.d.	0.003	n.d.	0.002	n.d.	0.004
	(epi)cat-ethyl-peonidin 3-O-Glc-2	n.d.	0.002	n.d.	0.003	n.d.	0.002	n.d.	0.004
	(epi)cat-ethyl-peonidin 3-O-Glc-3	n.d.	0.002	n.d.	0.003	n.d.	0.002	n.d.	0.004
	(epi)cat-ethyl-peonidin 3-O-Glc-4	n.d.	0.002	n.d.	0.003	n.d.	0.002	n.d.	0.004
	(epi)cat-ethyl-malvidin 3-O-Glc-1	n.d.	0.002	0.0003 ± 0.0006	0.002	0.0001 ± 0.0002	0.002	0.0002 ± 0.0003	0.004
	(epi)cat-ethyl-malvidin 3-O-Glc-2	n.d.	0.002	0.013 ± 0.002	0.002	0.001 ± 0.002	0.002	n.d.	0.004
	(epi)cat-ethyl-malvidin 3-O-Glc-3 and 4	n.d.	0.002	0.02 ± 0.006	0.002	0.001 ± 0.003	0.002	0.004 ± 0.007	0.004
	(epi)cat-ethyl-malvidin 3-O-coumaroyl-Glc (2 co-eluted isomers)	0.0005 ± 0.001	0.0005	0.03 ± 0.02	0.0008	0.0004 ± 0.0008	0.007	0.001 ± 0.002	0.0006
	Delphinidin 3-O-Glc-(epi)cat	n.d.	0.003	n.d.	0.003	n.d.	0.003	n.d.	0.005
	Cyanidin 3-O-Glc-(epi)cat	n.d.	0.004	0.003 ± 0.003	0.02	0.0003 ± 0.0005	0.004	n.d.	0.003
	Petunidin 3-O-Glc-(epi)cat	0.002 ± 0.003	0.03	0.015 ± 0.003	0.04	n.d.	0.03	0.004 ± 0.005	0.03

Table S1. *Cont.*

		5		6		7		8	
		**Conc. (mg·L^{-1})**	**LOQ (mg·L^{-1})**	**Conc. (mg·L^{-1})**	**LOQ (mg·L^{-1})**	**Conc. (mg·L^{-1})**	**LOQ (mg·L^{-1})**	**Conc. (mg·L^{-1})**	**LOQ (mg·L^{-1})**
Anthocyanins	*Peonidin 3-O-Glc-(epi)cat*	0.012 ± 0.009	0.003	0.02 ± 0.02	0.002	0.005 ± 0.003	0.002	0.009 ± 0.002	0.003
	Malvidin 3-O-Glc-(epi)cat (2 isomers)	0.092 ± 0.004	0.005	0.279 ± 0.008	0.02	0.03 ± 0.004	0.003	0.077 ± 0.008	0.002
	Malvidin 3-O-coumaroyl-Glc-(epi)cat (2 isomers)	0.0002 ± 0.0001	0.002	0.007 ± 0.003	0.03	0.001 ± 0.002	0.005	0.0011 ± 0.0005	0.002
	(epi)cat-delphinidin 3-O-Glc A-F bicyclic	n.d.	0.004	0.004 ± 0.002	0.0007	n.d.	0.003	n.d.	0.004
	(epi)cat-cyanidin 3-O-Glc A-F bicyclic	n.d.	0.002	n.d.	0.003	n.d.	0.004	n.d.	0.04
	(epi)cat-petunidin 3-O-Glc A-F bicyclic	n.d.	0.002	n.d.	0.002	n.d.	0.003	n.d.	0.005
	(epi)cat-peonidin 3-O-Glc A-F bicyclic	n.d.	0.004	0.001 ± 0.002	0.003	0.003 ± 0.006	0.007	n.d.	0.006
	(epi)cat-malvidin 3-O-Glc A-F bicyclic	0.001 ± 0.002	0.004	0.017 ± 0.009	0.06	0.004 ± 0.007	0.003	0.003 ± 0.004	0.002
	Caftaric-peonidin 3-O-Glc (2 isomers)	0.069 ± 0.01	0.002	0.011 ± 0.01	0.002	0.002 ± 0.002	0.0008	0.001 ± 0.001	0.003
	Caftaric-malvidin 3-O-Glc (2 isomers)	0.16 ± 0.02	0.002	0.044 ± 0.006	0.08	0.021 ± 0.003	0.003	0.04 ± 0.02	0.002
	Coutaric-malvidin 3-O-Glc (2 isomers)	n.d.	0.002	n.d.	0.002	n.d.	0.04	n.d.	0.03
	Pyranodelphinidin 3-O-Glc	n.d.	0.004	0.001 ± 0.002	0.002	n.d.	0.004	0.001 ± 0.002	0.03
	Pyranocyanidin 3-O-Glc	n.d.	0.02	n.d.	0.07	n.d.	0.02	n.d.	0.001
	Pyranopetunidin 3-O-Glc	n.d.	0.002	n.d.	0.006	0.0002 ± 0.0004	0.0005	0.0002 ± 0.0003	0.0007
	Pyranopeonidin 3-O-Glc	0.002 ± 0.002	0.003	0.004 ± 0.002	0.002	0.001 ± 0.002	0.004	0.004 ± 0.002	0.003
	Pyranomalvidin 3-O-Glc (vitisin B)	0.01 ± 0.002	0.02	0.073 ± 0.006	0.007	0.004 ± 0.002	0.003	0.048 ± 0.004	0.009
	Carboxypyranodelphinidin 3-O-Glc	n.d.	0.0008	0.016 ± 0.006	0.03	0.0001 ± 0.0001	0.009	0.012 ± 0.008	0.0008
	Carboxypyranocyanidin 3-O-Glc	n.d.	0.005	0.003 ± 0.003	0.02	n.d.	0.02	0.003 ± 0.003	0.02
	Carboxypyranopetunidin 3-O-Glc	0.0026 ± 0.0008	0.0008	0.008 ± 0.005	0.0007	n.d.	0.02	0.008 ± 0.005	0.03
	Carboxypyranopeonidin 3-O-Glc	0.015 ± 0.004	0.002	0.017 ± 0.003	0.002	0.0015 ± 0.0008	0.0005	0.013 ± 0.0005	0.0003
	Carboxypyranomalvidin 3-O-Glc (vitisin A)	0.131 ± 0.009	0.004	0.32 ± 0.03	0.005	0.032 ± 0.008	0.002	0.207 ± 0.007	0.005
	Pyranodelphinidin 3-O-acetyl-Glc	n.d.	0.0003	0.0001 ± 0.0001	0.0004	n.d.	0.004	n.d.	0.002
	Pyranocyanidin 3-O-acetyl-Glc	0.0002 ± 0.0004	0.0008	0.0004 ± 0.0004	0.002	n.d.	0.0006	0.0003 ± 0.0001	0.0007
	Pyranopetunidin 3-O-acetyl-Glc	n.d.	0.0005	n.d.	0.0008	0.0001 ± 0.0001	0.0007	n.d.	0.0003
	Pyranopeonidin 3-O-acetyl-Glc	n.d.	0.0006	n.d.	0.002	0.0001 ± 0.0002	0.002	n.d.	0.0008
	Pyranomalvidin 3-O-acetyl-Glc	0.001 ± 0.002	0.0003	0.03 ± 0.002	0.0002	n.d.	0.002	0.003 ± 0.005	0.002

Table S1. *Cont.*

		5		6		7		8	
		**Conc. (mg·L^{-1})**	**LOQ (mg·L^{-1})**	**Conc. (mg·L^{-1})**	**LOQ (mg·L^{-1})**	**Conc. (mg·L^{-1})**	**LOQ (mg·L^{-1})**	**Conc. (mg·L^{-1})**	**LOQ (mg·L^{-1})**
Anthocyanins	*Carboxypyranodelphinidin 3-O-acetyl-Glc*	0 ± 0.0001	0.0008	0.001 ± 0.002	0.0007	n.d.	0.0008	n.d.	0.0007
	Carboxypyranocyanidin 3-O-acetyl-Glc	0.0005 ± 0.0004	0.02	0.001 ± 0.0006	0.0006	0.0003 ± 0.0003	0.0006	0.0003 ± 0.0001	0.02
	Carboxypyranopetunidin 3-O-acetyl-Glc	n.d.	0.0006	0.0036 ± 0.0006	0.0002	n.d.	0.0006	0.001 ± 0.002	0.0003
	Carboxypyranopeonidin 3-O-acetyl-Glc	0.001 ± 0.002	0.004	0.03 ± 0.02	0.03	0.0009 ± 0.0009	0.008	0.01 ± 0.02	0.004
	Carboxypyranomalvidin 3-O-acetyl-Glc	0.007 ± 0.003	0.0006	0.17 ± 0.03	0.002	0.002 ± 0.003	0.0009	0.04 ± 0.02	0.002
	Pyranopetunidin 3-O-coumaroyl-Glc	n.d.	0.002	n.d.	0.0003	n.d.	0.0008	n.d.	0.0006
	Pyranopeonidin 3-O-coumaroyl-Glc	n.d.	0.002	0.005 ± 0.004	0.0003	n.d.	0.002	0.001 ± 0.002	0.002
	Pyranomalvidin 3-O-coumaroyl-Glc	n.d.	0.002	0.02 ± 0.02	0.002	n.d.	0.002	0.006 ± 0.005	0.0005
	Carboxypyranopetunidin 3-O-coumaroyl-Glc	n.d.	0.0008	0.002 ± 0.002	0.001	n.d.	0.001	0.001 ± 0.002	0.002
	Carboxypyranopeonidin 3-O-coumaroyl-Glc	0.004 ± 0.004	0.0007	0.012 ± 0.009	0.004	0.0005 ± 0.0006	0.0004	0.002 ± 0.003	0.0007
	Carboxypyranomalvidin 3-O-coumaroyl-Glc	0.007 ± 0.006	0.003	0.05 ± 0.03	0.002	0.004 ± 0.004	0.0003	0.012 ± 0.006	0.007
	p-hydroxyphenylpyranopeonidin 3-O-Glc	0.015 ± 0.01	0.04	0.0019 ± 0.0007	0.008	0.002 ± 0.003	0.0002	0.011 ± 0.006	0.02
	p-hydroxyphenylpyranomalvidin 3-O-Glc	0.13 ± 0.02	0.0003	0.07 ± 0.03	0.0003	0.034 ± 0.013	0.0003	0.14 ± 0.04	0.0002
	p-hydroxyphenylpyranopeonidin 3-O-acetyl-Glc	0.001 ± 0.001	0.0003	0.003 ± 0.002	0.0003	0.001 ± 0.002	0.0002	0.007 ± 0.004	0.0002
	p-hydroxyphenylpyranomalvidin 3-O-acetyl-Glc	0.008 ± 0.004	0.0002	0.03 ± 0.007	0.0004	0.011 ± 0.005	0.0004	0.033 ± 0.009	0.0002
	p-hydroxyphenylpyranopeonidin 3-O-coumaroyl-Glc	0.003 ± 0.002	0.0004	0.003 ± 0.001	0.0004	0.003 ± 0.003	0.0004	0.003 ± 0.002	0.0003
	p-hydroxyphenylpyranomalvidin 3-O-coumaroyl-Glc	0.03 ± 0.02	0.0003	0.016 ± 0.008	0.0002	0.015 ± 0.004	0.0002	0.021 ± 0.01	0.0003
	Catechylpyranopeonidin 3-O-Glc	0.023 ± 0.008	0.0002	0.008 ± 0.005	0.004	0.008 ± 0.001	0.0003	0.03 ± 0.02	0.002
	Catechylpyranomalvidin 3-O-Glc (pinotin A)	0.094 ± 0.021	0.0007	0.12 ± 0.02	0.0006	0.06 ± 0.005	0.0003	0.14 ± 0.03	0.0004
	Catechylpyranopetunidin 3-O-acetyl-Glc	0.001 ± 0.003	0.0002	0.002 ± 0.002	0.0004	n.d.	0.0004	0.002 ± 0.003	0.0003

Table S1. *Cont.*

		5		6		7		8	
		**Conc. (mg·L^{-1})**	**LOQ (mg·L^{-1})**	**Conc. (mg·L^{-1})**	**LOQ (mg·L^{-1})**	**Conc. (mg·L^{-1})**	**LOQ (mg·L^{-1})**	**Conc. (mg·L^{-1})**	**LOQ (mg·L^{-1})**
Anthocyanins	*Catechylpyranopeonidin 3-O-acetyl-Glc*	0.001 ± 0.002	0.0002	0.006 ± 0.005	0.0003	0.002 ± 0.001	0.0002	0.006 ± 0.004	0.0003
	Catechylpyranomalvidin 3-O-acetyl-Glc	0.004 ± 0.004	0.0005	0.083 ± 0.008	0.02	0.013 ± 0.005	0.0003	0.0366 ± 0.0004	0.0003
	Catechylpyranopetunidin 3-O-coumaroyl-Glc	0 ± 0.001	0.0002	0 ± 0.001	0.0002	0.0002 ± 0.0004	0.0002	0.002 ± 0.004	0.002
	Catechylpyranopeonidin 3-O-coumaroyl-Glc	0.004 ± 0.003	0.0002	0.002 ± 0.003	0.0005	0.0024 ± 0.0006	0.0004	0.009 ± 0.007	0.0002
	Catechylpyranomalvidin 3-O-coumaroyl-Glc	0.012 ± 0.006	0.002	0.028 ± 0.005	0.0003	0.034 ± 0.006	0.0002	0.04 ± 0.02	0.005
	Guaiacylpyranomalvidin 3-O-Glc	0.073 ± 0.008	0.0005	0.022 ± 0.003	0.006	0.02 ± 0.005	0.0005	0.06 ± 0.02	0.0007
	Guaiacylpyranomalvidin 3-O-acetyl-Glc	0.003 ± 0.002	0.0005	0.007 ± 0.003	0.0004	0.005 ± 0.003	0.0004	0.017 ± 0.006	0.0003
	Guaiacylpyranomalvidin 3-O-coumaroyl-Glc	0.008 ± 0.006	0.0004	0.003 ± 0.003	0.0004	0.007 ± 0.006	0.0004	0.005 ± 0.005	0.0004
	Syringylpyranomalvidin 3-O-Glc	0.0005 ± 0.0008	0.0008	n.d.	0.008	n.d.	0.0009	0.001 ± 0.001	0.002
	Petunidin 3-O-acetyl-Glc-(epi)cat	n.d.	0.004	n.d.	0.002	n.d.	0.003	n.d.	0.003
	Peonidin 3-O-acetyl-Glc-(epi)cat	n.d.	0.003	0.006 ± 0.005	0.004	n.d.	0.003	n.d.	0.003
	Malvidin 3-O-acetyl-Glc-(epi)cat	n.d.	0.002	0.04 ± 0.02	0.002	n.d.	0.0007	0.005 ± 0.005	0.0005
	Pyranopeonidin 3-O-Glc-(epi)cat	0.0012 ± 0.0004	0.0006	0.0011 ± 0.0001	0.0005	0.0003 ± 0.0001	0.0008	n.d.	0.002
	Pyranomalvidin 3-O-Glc-(epi)cat	0.003 ± 0.003	0.003	0.003 ± 0.003	0.0002	n.d.	0.0002	n.d.	0.0003
	Pyranomalvidin 3-O-coumaroyl-Glc-(epi)cat	0.004 ± 0.005	0.05	0 ± 0.001	0.0009	n.d.	0.002	n.d.	0.009
	Unknown "551"	0.001 ± 0.002	0.004	0.001 ± 0.002	0.002	0.0004 ± 0.0008	0.0004	0.004 ± 0.007	0.0006
	Unknown "581"	0.03 ± 0.02	0.002	0.04 ± 0.03	0.002	0.009 ± 0.008	0.002	0.09 ± 0.04	0.002
	Unknown "1095"	n.d.	0.02	n.d.	0.01	n.d.	0.03	n.d.	0.03
	Unknown "1099"	n.d.	0.01	0.002 ± 0.0007	< 0.002	0.0004 ± 0.0007	< 0.002	0.0008 ± 0.0008	< 0.002
Others (aminoacids and alcohols)	*Tyrosine*	10.4 ± 0.2	0.6	4.37 ± 0.07	0.2	5.91 ± 0.05	0.4	5.74 ± 0.06	0.3
	Tyrosol	7.85 ± 0.09	6	18.9 ± 0.5	0.9	15.01 ± 0.09	2	15.2 ± 0.3	2
	Hydroxytyrosol	1.1 ± 0.08	0.04	0.44 ± 0.05	0.03	0.07 ± 0.02	0.04	0.261 ± 0.005	0.03
	Tryptophol	0.4 ± 0.2	0.3	1.2 ± 0.5	0.2	0.3 ± 0.2	0.3	0.04 ± 0.03	0.2
	Tryptophan	1.4 ± 0.3	0	0.65 ± 0.1	0	0.4 ± 0.2	0	0.3 ± 0.2	0

Table S1. *Cont.*

		9		10		11		12	
		**Conc. (mg·L^{-1})**	**LOQ (mg·L^{-1})**	**Conc. (mg·L^{-1})**	**LOQ (mg·L^{-1})**	**Conc. (mg·L^{-1})**	**LOQ (mg·L^{-1})**	**Conc. (mg·L^{-1})**	**LOQ (mg·L^{-1})**
Benzoic acids and derivatives	*Gallic acid*	5.3 ± 0.2	0.03	3.43 ± 0.09	0.2	21.1 ± 0.5	0.1	1.03 ± 0.02	0.07
	Protocatechuic acid	3 ± 0.3	0.2	1.39 ± 0.03	0.07	0.71 ± 0.06	0.02	0.641 ± 0.003	0.03
	Syringic acid	1.6 ± 0.2	0.06	1.24 ± 0.04	0.03	0.29 ± 0.02	0.04	0.407 ± 0.006	0.05
	Vanillic acid	1.41 ± 0.07	0.2	1.1 ± 0.2	0.2	0.49 ± 0.02	0.2	0.4 ± 0.02	0.08
	Ethyl gallate	0.7 ± 0.3	0.02	0.5 ± 0.3	0.007	4 ± 0.4	0.008	0.14 ± 0.05	0.006
	Ethyl protocatechuate	0.5 ± 0.2	<0.02	0.2 ± 0.2	<0.02	0.06 ± 0.04	<0.02	0.07 ± 0.03	<0.02
Hydroxycinnamic acids and derivatives	*Caffeic acid*	2.5 ± 0.3	0.2	1.1 ± 0.1	0.03	2.1 ± 0.4	0.2	3.1 ± 0.3	0.08
	p-coumaric acid	2.8 ± 0.5	0.4	1 ± 0.2	0.2	0.52 ± 0.08	0.6	1.1 ± 0.3	0.3
	Ferulic acid	0.48 ± 0.07	0.04	0.3 ± 0.2	0.5	0.2 ± 0.2	0.04	0.2 ± 0.2	0.04
	Caftaric acid (cis- and trans- isomers)	6.4 ± 0.3	0.4	19.4 ± 0.6	0.3	16.4 ± 0.7	0.2	30.1 ± 0.5	0.3
	Coutaric acid (cis- and trans- isomers)	2.64 ± 0.04	0.04	14.4 ± 0.8	0.02	5.5 ± 0.5	0.09	7.1 ± 0.4	0.03
	Fertaric acid	3.49 ± 0.09	0.007	6.7 ± 0.2	0.006	4 ± 0.4	0.02	6.39 ± 0.07	0.009
	GRP (cis- and trans- isomers)	1.9 ± 0.5	0.3	23 ± 2	0.4	12 ± 2	0.05	20 ± 2	0.08
	Ethyl caffeate	0.67 ± 0.03	0.03	0.18 ± 0.03	0.007	0.68 ± 0.06	0.08	0.97 ± 0.09	0.07
	Ethyl coumarate	0.6 ± 0.2	0.03	0.24 ± 0.06	0.03	0.09 ± 0.03	0.02	0.18 ± 0.02	0.006
	trans-resveratrol	0.37 ± 0.05	0.05	0.228 ± 0.007	0.03	0.14 ± 0.03	0.02	0.112 ± 0.008	0.03
	cis-resveratrol	0.68 ± 0.03	0.05	0.17 ± 0.02	0.05	0.23 ± 0.02	0.02	0.12 ± 0.03	0.03
viniferin-1		0.06 ± 0.03	0.6	0.0233 ± 0.0007	0.09	0.1 ± 0.04	0. 2	0.1 ± 0.04	0.3
	viniferin-2	0.03 ± 0.02	0.05	0.02 ± 0.002	0.03	n.d.	0.02	0.023 ± 0.002	0.03
	trans-piceid	0.56 ± 0.08	0.7	0.33 ± 0.04	0.4	0.21 ± 0.03	0.8	0.19 ± 0.05	0.8
	cis-piceid	1.84 ± 0.1	0.2	1.4 ± 0.2	0.2	0.62 ± 0.06	0.05	0.52 ± 0.02	0.05
Flavonols	*Quercetin Glc*	0.01 ± 0.005	0.04	n.d.	0.02	0.008 ± 0.005	0.0009	n.d.	0.02
	Myricetin glucuronide	0.06 ± 0.02	0.3	0.15 ± 0.05	0.9	0.009 ± 0.004	0.03	0.001 ± 0.002	0.03
	Myricetin Glc	0.012 ± 0.007	0.005	0.08 ± 0.04	0.0008	0.003 ± 0.003	0.002	0.003 ± 0.002	0.006
	Quercetin glucuronide	0.008 ± 0.005	0.006	0.554 ± 0.009	0.002	0.1 ± 0.02	0.006	0.0055 ± 0.0006	0.0007
	Quercetin	n.d.	0.02	n.d.	0.2	n.d.	0.04	n.d.	0.03

Table S1. *Cont.*

		9		10		11		12	
		**Conc. (mg·L^{-1})**	**LOQ (mg·L^{-1})**	**Conc. (mg·L^{-1})**	**LOQ (mg·L^{-1})**	**Conc. (mg·L^{-1})**	**LOQ (mg·L^{-1})**	**Conc. (mg·L^{-1})**	**LOQ (mg·L^{-1})**
Flavan-3-ols	*Catechin*	8.4 ± 0.8	0.2	4.4 ± 0.3	0.08	5.8 ± 0.5	0.2	2.59 ± 0.05	0.04
	Galloylated dimer	n.d.	<0.002	n.d.	<0.002	n.d.	<0.002	n.d.	<0.002
	Trimer-1 (1 isomer)	0.2 ± 0.07	<0.007	0.08 ± 0.02	<0.007	0.002 ± 0.003	<0.007	0.002 ± 0.004	<0.007
	Trimers-2 (several isomers)	0.24 ± 0.04	0.04	0.08 ± 0.02	0.07	0.1 ± 0.03	0.05	0.0004 ± 0.0007	<0.002
	(epi)cat-ethyl-(epi)cat-1 (1 isomer)	n.d.	<0.08	n.d.	<0.08	n.d.	0.08	n.d.	<0.08
	(epi)cat-ethyl-(epi)cat-2 (2 isomers)	n.d.	0.002	n.d.	0.0007	n.d.	<0.0007	n.d.	0.02
	Epicatechin	7 ± 3	2	1.04 ± 0.05	0.6	3 ± 2	0.6	0.6 ± 0.2	0.2
	Dimer B2	2.37 ± 0.35	0.06	0.61 ± 0.04	0.02	0.3 ± 0.02	0.07	0.11 ± 0.03	0.03
	Dimer B1	9.22 ± 0.09	0.05	5.52 ± 0.07	0.02	0.99 ± 0.02	0.08	1.84 ± 0.05	0.02
	Dimer B3	1.23 ± 0.09	0.3	0.25 ± 0.03	0.2	0.16 ± 0.02	0.03	0.06 ± 0.02	0.03
	Dimer B4	1.8 ± 0.3	0.4	0.15 ± 0.1	0.4	0.106 ± 0.009	0.08	0.02 ± 0.02	0.06
Dihydroflavonols	*Astilbin*	0.31 ± 0.04	0.002	0.08 ± 0.03	0.006	0.61 ± 0.03	0.02	0.3 ± 0.02	0.003
	Taxifolin	0.5 ± 0.2	0.3	0.12 ± 0.02	0.4	0.32 ± 0.02	0.1	0.23 ± 0.02	0.2
Anthocyanins	*Malvidin 3,5-diGlc*	0.08 ± 0.02	2	0.08 ± 0.02	0.9	n.d.	0.03	n.d.	0.1
	Delphinidin 3,5-diGlc	n.d.	0.02	0.029 ± 0.003	0.02	n.d.	0.02	n.d.	0.02
	Cyanidin 3,5-diGlc	0.021 ± 0.004	0.04	0.0175 ± 0.0004	0.02	n.d.	0.02	n.d.	0.02
	Petunidin 3,5-diGlc	n.d.	0.02	n.d.	0.03	n.d.	0.02	n.d.	0.02
	Peonidin 3,5-diGlc	0.05 ± 0.02	0.3	0.043 ± 0.006	0.2	n.d.	0.02	n.d.	0.04
	Malvidin 3-O-Glc	13.3 ± 0.3	0.06	11.8 ± 0.2	0.05	0.079 ± 0.007	0.005	0.731 ± 0.007	0.02
	Delphinidin 3-O-Glc	0.19 ± 0.007	0.003	0.422 ± 0.009	0.006	0.0018 ± 0.0009	0.004	0.0014 ± 0.0007	0.002
	Cyanidin 3-O-Glc	0.0395 ± 0.0009	0.003	0.031 ± 0.004	0.003	0.0004 ± 0.0001	0.0008	0.00144 ± 0.00007	0.002
	Petunidin 3-O-Glc	0.78 ± 0.1	0.03	1.61 ± 0.07	0.05	0.0022 ± 0.0008	0.01	0.013 ± 0.003	0.0006
	Peonidin 3-O-Glc	1.04 ± 0.02	0.02	0.43 ± 0.02	0.03	0.015 ± 0.002	0.003	0.074 ± 0.01	0.004
	Delphinidin 3-O-acetyl-Glc	0.094 ± 0.003	0.004	0.085 ± 0.006	0.001	0.0004 ± 0.0005	0.0008	0.0004 ± 0.0004	0.002
	Cyanidin 3-O-acetyl-Glc	0.0731 ± 0.0006	0.003	0.037 ± 0.002	0.0009	n.d.	0.002	0.0014 ± 0.0003	0.0006
	Petunidin 3-O-acetyl-Glc	0.209 ± 0.005	0.002	0.1281 ± 0.0005	0.003	0.0002 ± 0.0005	0.002	0.0028 ± 0.0004	0.0008
	Peonidin 3-O-acetyl-Glc	1.23 ± 0.03	0.002	0.222 ± 0.008	0.0009	0 ± 0.001	0.0003	0.059 ± 0.003	0.0007

Table S1. *Cont.*

		9		10		11		12	
		Conc. (mg·L^{-1})	LOQ (mg·L^{-1})	Conc. (mg·L^{-1})	LOQ (mg·L^{-1})	Conc. (mg·L^{-1})	LOQ (mg·L^{-1})	Conc. (mg·L^{-1})	LOQ (mg·L^{-1})
Anthocyanins	*Malvidin 3-O-acetyl-Glc*	6 ± 0.2	0.02	1.29 ± 0.03	0.01	0.0012 ± 0.0005	0.002	0.169 ± 0.004	0.003
	Delphinidin 3-O-coumaroyl-Glc (cis- and trans- isomers)	0.021 ± 0.008	0.008	0.14 ± 0.03	0.009	0.001 ± 0.003	0.0006	0.0002 ± 0.0004	0.002
	Cyanidin 3-O-coumaroyl-Glc (cis- and trans- isomers)	0.029 ± 0.008	0.003	0.08 ± 0.02	0.004	n.d.	0.0004	0.001 ± 0.002	0.0004
	Petunidin 3-O-coumaroyl-Glc (cis- and trans- isomers)	0.051 ± 0.003	0.0006	0.21 ± 0.02	0.009	0.001 ± 0.002	0.0002	0.0021 ± 0.001	0.0003
	Peonidin 3-O-coumaroyl-Glc (cis- and trans- isomers)	0.23 ± 0.05	0.006	0.32 ± 0.02	0.005	0.0005 ± 0.0002	0.0003	0.025 ± 0.005	0.002
	Malvidin 3-O-coumaroyl-Glc (cis- and trans- isomers)	1.29 ± 0.02	0.04	1.96 ± 0.05	0.02	0.003 ± 0.002	0.0006	0.079 ± 0.002	0.002
	Delphinidin 3-O-caffeoyl-Glc	n.d.	0.003	n.d.	0.003	n.d.	0.003	n.d.	0.003
	Cyanidin 3-O-caffeoyl-Glc	0.003 ± 0.005	0.001	n.d.	0.0008	n.d.	0.003	n.d.	0.0007
	Petunidin 3-O-caffeoyl-Glc	n.d.	0.002	n.d.	0.002	n.d.	0.0006	n.d.	0.002
	Peonidin 3-O-caffeoyl-Glc	0.04 ± 0.03	0.006	0.018 ± 0.01	0.0007	n.d.	0.003	n.d.	0.002
	Malvidin 3-O-caffeoyl-Glc	0.09 ± 0.04	0.05	0.075 ± 0.009	0.002	n.d.	0.0009	0.0003 ± 0.0006	0.002
	(epi)cat-ethyl-peonidin 3-O-Glc-1	n.d.	0.004	n.d.	0.0008	n.d.	0.003	n.d.	0.005
	(epi)cat-ethyl-peonidin 3-O-Glc-2	n.d.	0.004	n.d.	0.0008	n.d.	0.003	n.d.	0.005
	(epi)cat-ethyl-peonidin 3-O-Glc-3	n.d.	0.004	n.d.	0.0008	0.002 ± 0.003	0.003	n.d.	0.005
	(epi)cat-ethyl-peonidin 3-O-Glc-4	n.d.	0.004	n.d.	0.0008	n.d.	0.003	n.d.	0.005
	(epi)cat-ethyl-malvidin 3-O-Glc-1	n.d.	0.002	0.00005 ± 0.00009	0.002	0.001 ± 0.002	0.002	n.d.	0.004
	(epi)cat-ethyl-malvidin 3-O-Glc-2	n.d.	0.002	0.014 ± 0.006	0.002	0.012 ± 0.009	0.002	n.d.	0.004
	(epi)cat-ethyl-malvidin 3-O-Glc-3 and 4	0.01 ± 0.02	0.002	0.051 ± 0.008	0.002	0.03 ± 0.03	0.002	n.d.	0.004
	(epi)cat-ethyl-malvidin 3-O-coumaroyl-Glc (2 co-eluted isomers)	0.003 ± 0.005	0.005	0.02 ± 0.01	0.0005	0.001 ± 0.002	0.02	n.d.	0.0006
	Delphinidin 3-O-Glc-(epi)cat	n.d.	0.002	n.d.	0.002	n.d.	0.004	n.d.	0.004

Table S1. *Cont.*

		9		10		11		12	
		**Conc. (mg·L^{-1})**	**LOQ (mg·L^{-1})**	**Conc. (mg·L^{-1})**	**LOQ (mg·L^{-1})**	**Conc. (mg·L^{-1})**	**LOQ (mg·L^{-1})**	**Conc. (mg·L^{-1})**	**LOQ (mg·L^{-1})**
Anthocyanins	*Cyanidin 3-O-Glc-(epi)cat*	0.001 ± 0.002	0.02	0.02 ± 0.02	0.0008	n.d.	0.002	n.d.	0.0009
	Petunidin 3-O-Glc-(epi)cat	0.01 ± 0.004	0.07	0.009 ± 0.01	0.06	n.d.	0.0007	n.d.	0.004
	Peonidin 3-O-Glc-(epi)cat	0.04 ± 0.02	0.002	0.03 ± 0.02	0.002	n.d.	0.002	0.003 ± 0.005	0.006
	Malvidin 3-O-Glc-(epi)cat (2 isomers)	0.34 ± 0.04	0.02	0.14 ± 0.02	0.02	0.0007 ± 0.0002	0.003	0.004 ± 0.003	0.002
	Malvidin 3-O-coumaroyl-Glc-(epi)cat (2 isomers)	0.001 ± 0.002	0.006	0.0013 ± 0.0006	0.006	n.d.	0.0006	n.d.	0.003
	(epi)cat-delphinidin 3-O-Glc A-F bicyclic	n.d.	0.004	n.d.	0.005	n.d.	0.003	n.d.	0.004
	(epi)cat-cyanidin 3-O-Glc A-F bicyclic	n.d.	0.008	n.d.	0.002	n.d.	0.004	n.d.	0.004
	(epi)cat-petunidin 3-O-Glc A-F bicyclic	0.001 ± 0.002	0.002	0.002 ± 0.002	0.002	n.d.	0.003	n.d.	0.003
	(epi)cat-peonidin 3-O-Glc A-F bicyclic	0.003 ± 0.004	0.002	n.d.	0.003	n.d.	0.005	n.d.	0.003
	(epi)cat-malvidin 3-O-Glc A-F bicyclic	0.005 ± 0.005	0.05	0.01 ± 0.02	0.03	0.001 ± 0.002	0.03	n.d.	0.003
	Caftaric-peonidin 3-O-Glc (2 isomers)	0.008 ± 0.006	0.003	n.d.	0.02	n.d.	0.001	0.001 ± 0.003	0.002
	Caftaric-malvidin 3-O-Glc (2 isomers)	0.019 ± 0.007	0.003	0.012 ± 0.004	0.002	n.d.	0.002	0.007 ± 0.003	0.002
	Coutaric-malvidin 3-O-Glc (2 isomers)	n.d.	0.003	n.d.	0.003	n.d.	0.002	n.d.	0.004
	Pyranodelphinidin 3-O-Glc	n.d.	0.03	0.002 ± 0.003	0.003	0.0003 ± 0.0006	0.004	n.d.	0.03
	Pyranocyanidin 3-O-Glc	n.d.	0.007	n.d.	0.04	n.d.	0.04	n.d.	0.01
	Pyranopetunidin 3-O-Glc	0.002 ± 0.002	0.0006	0.006 ± 0.002	0.01	0.0003 ± 0.0006	0.01	n.d.	0.003
	Pyranopeonidin 3-O-Glc	0.003 ± 0.002	0.006	0.006 ± 0.002	0.003	0.0061 ± 0.0006	0.002	0.0009 ± 0.0002	0.002
	Pyranomalvidin 3-O-Glc (vitisin B)	0.0384 ± 0.001	0.02	0.181 ± 0.006	0.02	0.048 ± 0.004	0.004	0.005 ± 0.002	0.002
	Carboxypyranodelphinidin 3-O-Glc	0.001 ± 0.002	0.02	0.011 ± 0.007	0.03	0.001 ± 0.001	0.02	0.001 ± 0.002	0.02
	Carboxypyranocyanidin 3-O-Glc	0.002 ± 0.004	0.02	0.003 ± 0.002	0.02	0.001 ± 0.002	0.0005	n.d.	0.003
	Carboxypyranopetunidin 3-O-Glc	0.002 ± 0.002	0.02	0.004 ± 0.004	0.02	0.002 ± 0.002	0.004	0.002 ± 0.004	0.002
	Carboxypyranopeonidin 3-O-Glc	0.013 ± 0.004	0.002	0.0046 ± 0.0005	0.0003	0.003 ± 0.0002	0.0005	0.0017 ± 0.0007	0.0004
	Carboxypyranomalvidin 3-O-Glc (vitisin A)	0.2 ± 0.02	0.003	0.17 ± 0.02	0.005	0.06 ± 0.02	0.002	0.012 ± 0.004	0.0003
	Pyranodelphinidin 3-O-acetyl-Glc	n.d.	0.0005	n.d.	0.0003	n.d.	0.005	n.d.	0.0002
	Pyranocyanidin 3-O-acetyl-Glc	0.0002 ± 0.0005	0.0009	n.d.	0.0009	n.d.	0.0006	n.d.	0.0005

Table S1. *Cont.*

		9		10		11		12	
		**Conc. (mg·L^{-1})**	**LOQ (mg·L^{-1})**	**Conc. (mg·L^{-1})**	**LOQ (mg·L^{-1})**	**Conc. (mg·L^{-1})**	**LOQ (mg·L^{-1})**	**Conc. (mg·L^{-1})**	**LOQ (mg·L^{-1})**
Anthocyanins	*Pyranopetunidin 3-O-acetyl-Glc*	n.d.	0.0005	n.d.	0.0005	n.d.	0.0007	n.d.	0.003
	Pyranopeonidin 3-O-acetyl-Glc	0.002 ± 0.004	0.0008	0.0003 ± 0.0005	0.003	n.d.	0.0006	n.d.	0.0005
	Pyranomalvidin 3-O-acetyl-Glc	0.009 ± 0.005	0.0008	0.01 ± 0.006	0.0004	0.0002 ± 0.0003	0.0009	n.d.	0.0007
	Carboxypyranodelphinidin 3-O-acetyl-Glc	n.d.	0.02	0.0003 ± 0.0005	0.0007	n.d.	0.002	n.d.	0.0006
	Carboxypyranocyanidin 3-O-acetyl-Glc	0.0004 ± 0.0003	0.0004	0.0008 ± 0.0008	0.0009	n.d.	0.0005	n.d.	0.002
	Carboxypyranopetunidin 3-O-acetyl-Glc	n.d.	0.0004	n.d.	0.008	n.d.	0.0007	n.d.	0.0003
	Carboxypyranopeonidin 3-O-acetyl-Glc	0.005 ± 0.005	0.02	n.d.	0.008	n.d.	0.002	0.001 ± 0.002	0.003
	Carboxypyranomalvidin 3-O-acetyl-Glc	0.08 ± 0.03	0.02	0.007 ± 0.01	0.001	0.0005 ± 0.0005	0.002	0.0004 ± 0.0008	0.0006
	Pyranopetunidin 3-O-coumaroyl-Glc	n.d.	0.003	n.d.	0.002	n.d.	0.0004	n.d.	0.0008
	Pyranopeonidin 3-O-coumaroyl-Glc	0.001 ± 0.001	0.0005	0.005 ± 0.005	0.0005	n.d.	0.003	n.d.	0.0007
	Pyranomalvidin 3-O-coumaroyl-Glc	0.003 ± 0.003	0.0007	0.041 ± 0.007	0.002	n.d.	0.0008	0.002 ± 0.003	0.0007
	Carboxypyranopetunidin 3-O-coumaroyl-Glc	n.d.	0.0007	n.d.	0.002	n.d.	0.0007	n.d.	0.004
	Carboxypyranopeonidin 3-O-coumaroyl-Glc	0.002 ± 0.004	0.001	0.003 ± 0.004	0.0005	n.d.	0.001	n.d.	0.0005
	Carboxypyranomalvidin 3-O-coumaroyl-Glc	0.009 ± 0.008	0.005	0.012 ± 0.004	0.002	0.004 ± 0.004	0.0004	0.003 ± 0.005	0.003
	p-hydroxyphenylpyranopeonidin 3-O-Glc	0.018 ± 0.002	0.008	0.006 ± 0.003	0.002	0.021 ± 0.004	0.0005	0.015 ± 0.002	0.0003
	p-hydroxyphenylpyranomalvidin 3-O-Glc	0.26 ± 0.03	0.004	0.25 ± 0.03	0.0003	0.12 ± 0.03	0.0003	0.11 ± 0.04	0.0002
	p-hydroxyphenylpyranopeonidin 3-O-acetyl-Glc	0.012 ± 0.005	0.0003	0.002 ± 0.002	0.0002	n.d.	0.0004	0.005 ± 0.004	0.0003
	p-hydroxyphenylpyranomalvidin 3-O-acetyl-Glc	0.08 ± 0.02	0.0002	0.01 ± 0.003	0.002	0.0005 ± 0.0009	0.0002	0.028 ± 0.007	0.0003
	p-hydroxyphenylpyranopeonidin 3-O-coumaroyl-Glc	0.002 ± 0.001	0.0003	0.01 ± 0.02	0.008	n.d.	0.0003	0.013 ± 0.003	0.0006

Table S1. *Cont.*

		9		10		11		12	
		Conc. (mg·L^{-1})	LOQ (mg·L^{-1})	Conc. (mg·L^{-1})	LOQ (mg·L^{-1})	Conc. (mg·L^{-1})	LOQ (mg·L^{-1})	Conc. (mg·L^{-1})	LOQ (mg·L^{-1})
Anthocyanins	*p-hydroxyphenylpyranomalvidin 3-O-coumaroyl-Glc*	0.06 ± 0.02	0.0004	0.07 ± 0.02	0.0009	0.003 ± 0.003	0.0005	0.044 ± 0.005	0.002
	Catechylpyranopeonidin 3-O-Glc	0.021 ± 0.006	0.003	0.025 ± 0.008	0.0002	0.015 ± 0.01	0.0003	0.01 ± 0.002	0.0002
	Catechylpyranomalvidin 3-O-Glc (pinotin A)	0.07 ± 0.02	0.0004	0.072 ± 0.009	0.0002	0.039 ± 0.009	0.001	0.11 ± 0.03	0.0002
	Catechylpyranopetunidin 3-O-acetyl-Glc	0.004 ± 0.005	0.0002	0.0006 ± 0.0008	0.0004	n.d.	0.0005	0.0004 ± 0.0004	0.0003
	Catechylpyranopeonidin 3-O-acetyl-Glc	0.005 ± 0.004	0.0003	0.003 ± 0.003	0.0004	n.d.	0.0003	0.004 ± 0.004	0.0004
	Catechylpyranomalvidin 3-O-acetyl-Glc	0.032 ± 0.009	0.0004	0.006 ± 0.008	0.0007	0.0003 ± 0.0005	0.0004	0.025 ± 0.008	0.005
	Catechylpyranopetunidin 3-O-coumaroyl-Glc	n.d.	0.0005	0.0014 ± 0.0009	0.0005	n.d.	0.0002	0.003 ± 0.002	0.0003
	Catechylpyranopeonidin 3-O-coumaroyl-Glc	0.0011 ± 0.0006	0.0002	0.008 ± 0.002	0.0003	0.001 ± 0.003	0.0003	0.008 ± 0.003	0.0003
	Catechylpyranomalvidin 3-O-coumaroyl-Glc	0.011 ± 0.005	0.002	0.021 ± 0.003	0.004	n.d.	0.0003	0.024 ± 0.004	0.0009
	Guaiacylpyranomalvidin 3-O-Glc	0.06 ± 0.007	0.004	0.08 ± 0.03	0.0004	0.04 ± 0.02	0.0004	0.11 ± 0.03	0.0004
	Guaiacylpyranomalvidin 3-O-acetyl-Glc	0.015 ± 0.006	0.003	0.003 ± 0.005	0.0003	n.d.	0.0005	0.022 ± 0.005	0.0003
	Guaiacylpyranomalvidin 3-O-coumaroyl-Glc	0.007 ± 0.004	0.002	0.015 ± 0.006	0.0002	0.0004 ± 0.0008	0.0003	0.016 ± 0.007	0.0005
	Syringylpyranomalvidin 3-O-Glc	0.002 ± 0.003	0.0006	0.003 ± 0.002	0.002	n.d.	0.0009	n.d.	0.002
	Petunidin 3-O-acetyl-Glc-(epi)cat	n.d.	0.003	n.d.	0.003	n.d.	0.02	n.d.	0.002
	Peonidin 3-O-acetyl-Glc-(epi)cat	0.0004 ± 0.0008	0.002	n.d.	0.004	n.d.	0.004	n.d.	0.002
	Malvidin 3-O-acetyl-Glc-(epi)cat	0.02 ± 0.02	0.002	0.004 ± 0.007	0.0008	n.d.	0.0007	n.d.	0.0005
	Pyranopeonidin 3-O-Glc-(epi)cat	0.0008 ± 0.0008	0.0007	0.0008 ± 0.0004	0.002	0.0082 ± 0.0007	0.001	n.d.	0.002
	Pyranomalvidin 3-O-Glc-(epi)cat	0.0007 ± 0.0007	0.0002	n.d.	0.0007	0.05 ± 0.005	0.0004	n.d.	0.0002
	Pyranomalvidin 3-O-coumaroyl-Glc-(epi)cat	n.d.	0.0009	0.003 ± 0.005	0.0009	0 ± 0.001	0.002	n.d.	0.002

Table S1. *Cont.*

		9		10		11		12	
		**Conc. (mg·L^{-1})**	**LOQ (mg·L^{-1})**	**Conc. (mg·L^{-1})**	**LOQ (mg·L^{-1})**	**Conc. (mg·L^{-1})**	**LOQ (mg·L^{-1})**	**Conc. (mg·L^{-1})**	**LOQ (mg·L^{-1})**
Anthocyanins	*Unknown "551"*	0.0005 ± 0.0003	0.003	0.0004 ± 0.0003	0.04	0.002 ± 0.003	0.0007	n.d.	0.002
	Unknown "581"	0.024 ± 0.006	0.003	0.072 ± 0.007	0.003	0.006 ± 0.004	0.02	0.007 ± 0.007	0.004
	Unknown "1095"	n.d.	0.02	n.d.	0.02	0.011 ± 0.006	0.02	n.d.	0.02
	Unknown "1099"	0.00135 ± 0.00003	0.002	0.001 ± 0.002	0.03	n.d.	<0.002	n.d.	<0.002
Others (aminoacids and alcohols)	*Tyrosine*	9 ± 0.08	0.6	6.59 ± 0.07	0.3	14 ± 0.3	0.9	10.92 ± 0.07	1
	Tyrosol	16.07 ± 0.34	4	8 ± 0.2	3	7.3 ± 0.2	0.6	7.1 ± 0.2	0.7
	Hydroxytyrosol	0.95 ± 0.08	0.03	0.2 ± 0.03	0.05	0.82 ± 0.08	0.04	0.1248 ± 0.0005	0.04
	Tryptophol	1.39 ± 0.28	0.4	4.4 ± 0.4	0.3	n.d.	0.4	0.4 ± 0.3	0.34
	Tryptophan	1.65 ± 0.38	0	2.03 ± 0.02	0	n.d.	0	1 ± 0.2	0

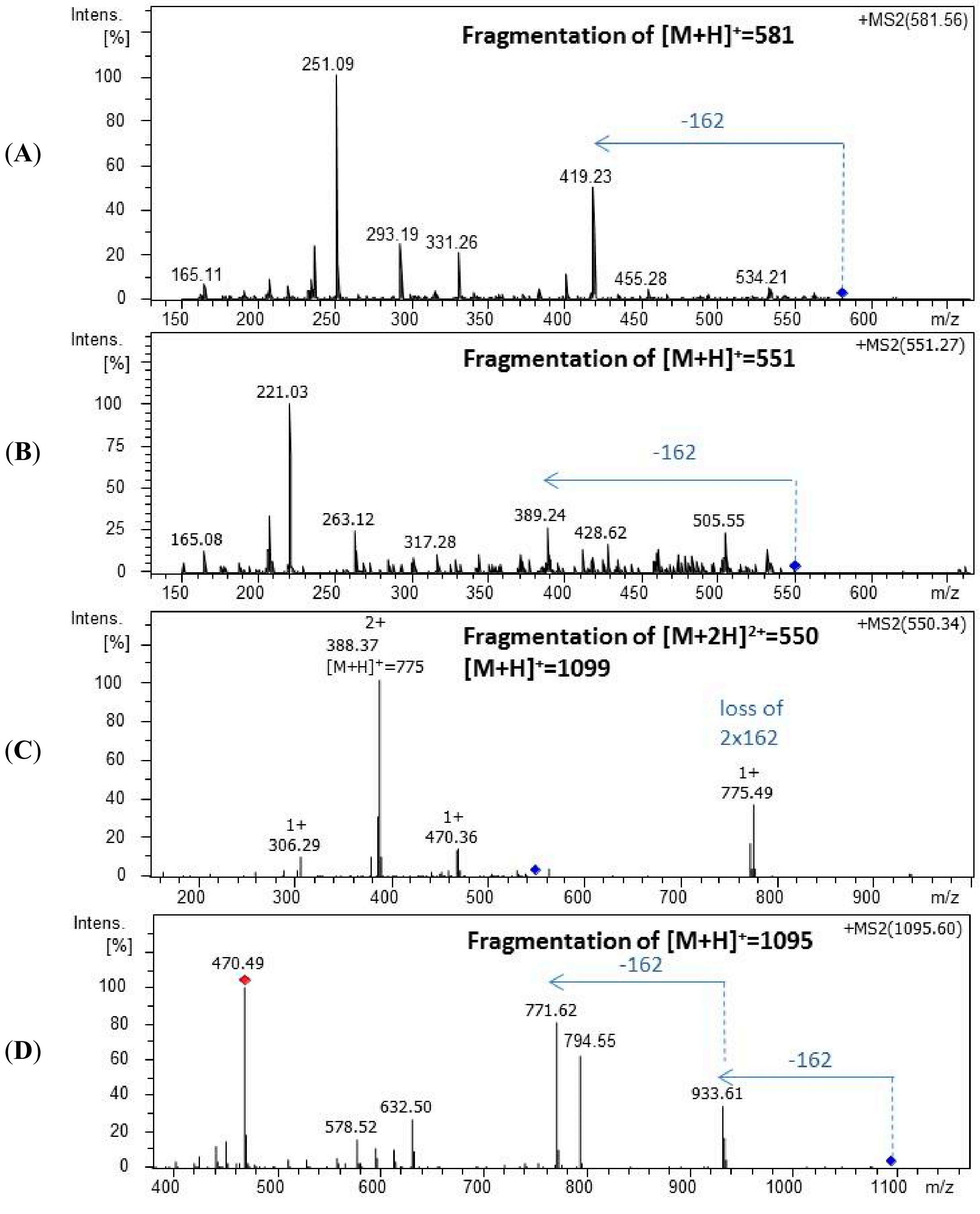

Figure S1. Ion trap MS/MS fragmentation spectra (ESI+ mode) of (**A**) "unknown581", (**B**) "unknown551", (**C**) "unknown1099" (from the doubly charged parent ion at *m/z* 550 Th) and (**D**) "unknown1095".
